# Synthetic and biosynthetic methods for selective cyclisations of 4,5-epoxy alcohols to tetrahydropyrans

**DOI:** 10.1039/d1ob01905h

**Published:** 2022-01-14

**Authors:** James I. Bowen, Luoyi Wang, Matthew P. Crump, Christine L. Willis

**Affiliations:** School of Chemistry, University of Bristol Cantock's Close Bristol BS8 1TS UK chris.willis@bristol.ac.uk; CAS Key Laboratory of Microbial Physiological and Metabolic Engineering, State Key Laboratory of Microbial Resources, Institute of Microbiology, Chinese Academy of Sciences Beijing China

## Abstract

Tetrahydropyrans (THPs) are common structural motifs found in natural products and synthetic therapeutic molecules. In Nature these 6-membered oxygen heterocycles are often assembled *via* intramolecular reactions involving either oxy-Michael additions or ring opening of epoxy-alcohols. Indeed, the polyether natural products have been particularly widely studied due to their fascinating structures and important biological properties; these are commonly formed *via endo*-selective epoxide-opening cascades. In this review we outline synthetic approaches for *endo*-selective intramolecular epoxide ring opening (IERO) of 4,5-epoxy-alcohols and their applications in natural product synthesis. In addition, the biosynthesis of THP-containing natural products which utilise IERO reactions are reviewed.

## Abstract 2

In this review, methods for the selective intramolecular epoxide ring opening (IERO) of 4,5-epoxy-alcohols are discussed as well as biosynthetic pathways to tetrahydropyran-containing natural products which utilise IERO reactions.

## Graphical abstract

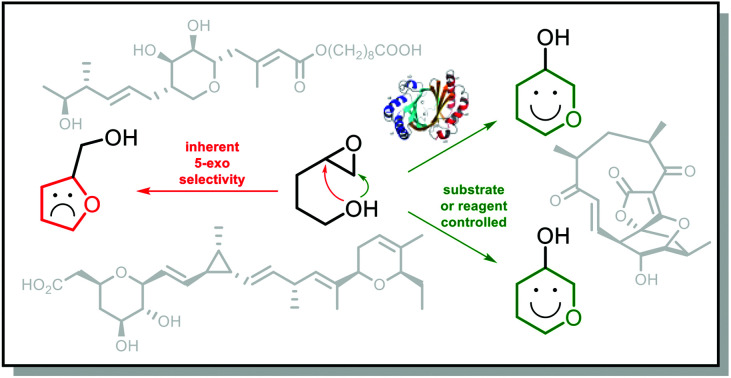

## 1. Introduction

**Fig. 1:**
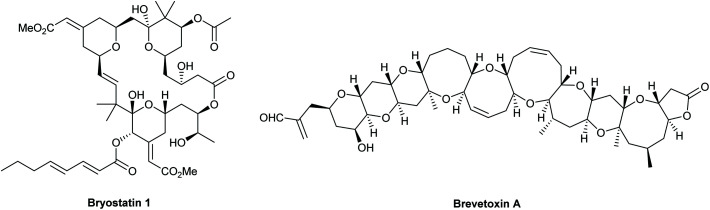
Bryostatin 1 and brevetoxin A.

Tetrahydropyrans (THPs) are common structural features in many classes of natural products and biologically active molecules such as the potent anti-cancer macrolide lactone bryostatin 1, and various marine polycyclic polyethers including the brevotoxins (Fig. 1). Furthermore, these rings are regularly employed as scaffolds in medicinal chemistry programs and indeed they are the sixth most prevalent ring system amongst all FDA approved small molecule drugs.^1^

**Fig. 2:**
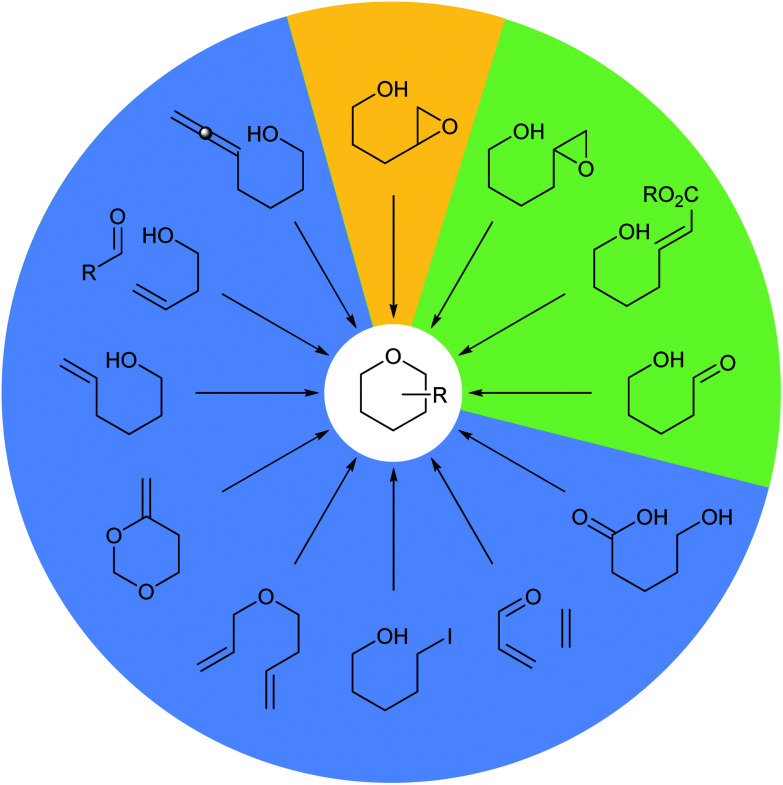
Common methods for the synthesis and biosynthesis of THP rings. Synthetic methods (blue), synthetic and biosynthetic methods (green) and the focus of this review (yellow).

**Fig. 3:**
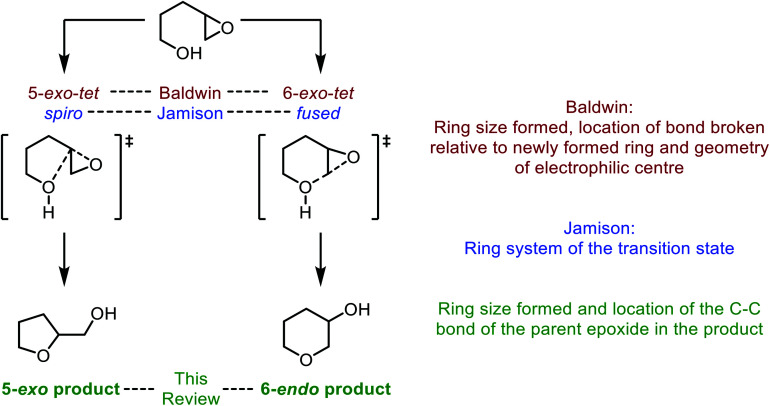
Intramolecular epoxide ring opening of 4,5-epoxy alcohols and corresponding nomenclature as described by Baldwin (red), Jamison (blue) and in this review (green).

Many methods have been developed for the synthesis of THPs as described in previous informative reviews.^2–6^ Common approaches include Prins cyclisations, hetero-Diels–Alder reactions and ring closing metatheses (Fig. 2).^7–9^ In addition, reviews dedicated to the synthesis of natural products with structures incorporating THP rings have been published.^10–13^ As well as synthetic studies, the biosynthesis of THP-containing natural products is of widespread interest and recent work has revealed three main processes found in Nature: oxa-Michael conjugate addition, modification of hemiacetals and intramolecular nucleophilic opening of epoxides (Fig. 2).^14–16^ Indeed, intramolecular epoxide ring opening (IERO) of 4,5-epoxy alcohols has also been widely used in organic synthesis to create oxygen heterocycles giving either 5-membered tetrahydrofurans (THFs) or 6-membered THPs depending on the site of attack of the alcohol onto the epoxide (Fig. 3). This review focuses on the synthesis and biosynthesis of THP rings *via* IERO of 4,5-epoxy alcohols, highlighting how regioselectivity of epoxide ring opening may be achieved.

In the literature the terms 5-*exo* and 6-*endo* have often been used to describe formation of THFs and THPs respectively from 4,5-epoxy alcohols.^17^ This nomenclature is adopted in this review and refers to the process of intramolecular epoxide ring opening by a numerical prefix, describing the size of the ring formed, followed by *exo*/*endo* to indicate whether the C–C bond of the initial epoxide is located outside or inside the newly formed ring respectively. It is important to note that this nomenclature does not relate to Baldwin's rules, a set of guidelines for predicting the outcome of ring-forming reactions whereby the numerical prefix defines the ring size formed, *exo* and *endo* refer to whether the bond broken during cyclisation is inside or outside of the newly formed ring, and the suffix indicates the geometry of the electrophile. Using these rules both modes of cyclisation of 4,5-epoxy alcohols are *exo* processes as the C–O bond broken during ring formation is outside of the newly formed heterocycle (Fig. 3).^18,19^ According to Baldwin's rules, both 5-*exo-tet* and 6-*exo-tet* are favoured processes, whilst 6-*endo-tet* cyclisations are disfavoured. Jamison *et al.* have suggested an alternative nomenclature which refers to the transition state of the cyclisation as either spiro or fused for THF and THP formation respectively (Fig. 3).^5^

Whilst both THF and THP rings can be generated from 4,5-epoxy alcohols, the THF is often the major product as noted by Baldwin.^17^ This selectivity originates from improved orbital overlap and a lower overall energetic barrier for formation in a 5-*exo* process.^20^ Furthermore, formation of 5-membered rings is often preferred kinetically over the corresponding 6-membered rings. Therefore, to form THP rings through intramolecular epoxide ring opening of 4,5-epoxy alcohols, this inherent preference to form the 5-membered ring needs to be overcome. Many synthetic methods have been developed to favour THP formation using substrates and reaction conditions which either stabilise the 6-*endo* transition state or destabilise the 5-*exo* transition state. To achieve selectivity in natural product biosynthesis, enzymes are often used. In this review, we will outline synthetic strategies which have been developed to favour THP formation alongside examples of their use in total synthesis. This topic will be divided into substrate controlled and reagent controlled cyclisations with examples of their use in natural product synthesis. Furthermore, examples of THP and THF formation in natural product biosynthesis will be described, highlighting how nature employs enzymes to promote and control IERO.

## 2. Synthetic methods

Vilotijevic and Jamison have published reviews on the use of epoxide-opening cascades to create ladder polyethers and oxasqualenoid natural products.^5,21^ In this section of our review, we build upon these publications providing an overview of synthetic approaches to achieve 6-*endo*-selective epoxide ring opening of 4,5-epoxy alcohols alongside their use in natural product synthesis.

### 2.1 Substrate controlled formation of THPs from 4,5-epoxy alcohols

#### 2.1.1 Alkenic epoxides

**Scheme 1:**
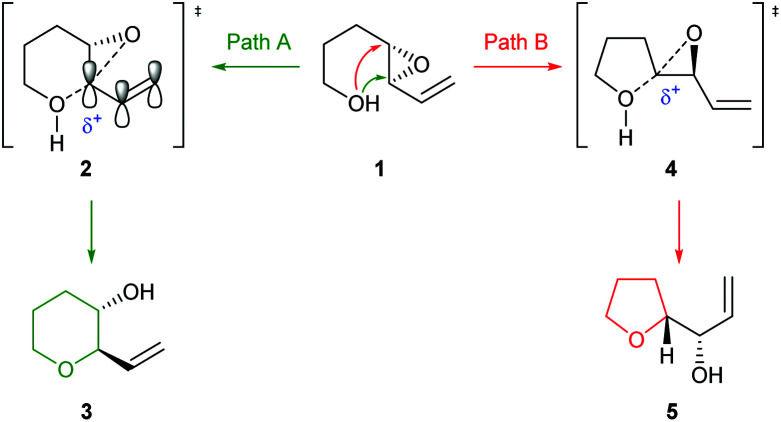
Stabilisation of 6-*endo* transition state by conjugation.

Due to widespread interest in marine polycyclic polyether natural products, such as the ladder polyether gambierol and the oxasqualenoid thyrsiferol, many groups have developed methods to achieve selective 6-*endo* ring closure of 4,5-epoxy alcohols. In 1985 Nicolaou reported studies towards the total synthesis of brevotoxin B in which selective cyclisation could be achieved by stabilisation of the incipient positive charge in the 6-*endo* transition state through the incorporation of a π-system adjacent to the epoxide (Scheme 1).^22^

**Scheme 2:**
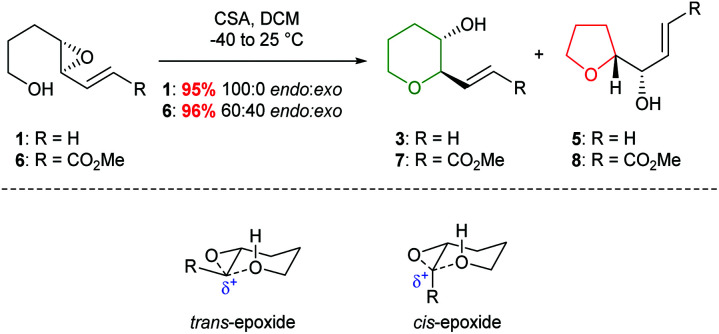
Acid catalysed cyclisation of unsaturated epoxides 1 and 6 (top) and transition states for the cyclisation of *cis* and *trans*-epoxides (bottom).

Indeed, acid catalysed cyclisation of unsaturated epoxide 1 proceeded with complete regioselectivity, affording 6-*endo* product 3 in excellent yield (Scheme 2).^23^ Various π-systems have been investigated including, vinyl halides and unsaturated esters, where the greater electron withdrawing nature of the conjugated functional group decreases the observed selectivity. Whilst *trans*-epoxides often give good yields of THPs under these conditions, poor regioselectivity was obtained with the corresponding *cis*-epoxides. Nicolaou proposed that *cis*-epoxides are unable to adopt the required planar geometry to achieve stabilisation in the transition state. Indeed, earlier studies by Coxon *et al.* illustrated that cyclisation of *cis*-4,5-epoxy alcohols gave preferential 5-*exo* ring closure compared to the corresponding *trans*-epoxides.^24^ The 6-*endo* transition state for *cis*-epoxides requires a group to be placed axially, resulting in steric clashes and an overall increased barrier for THP formation (Scheme 2).

**Scheme 3:**
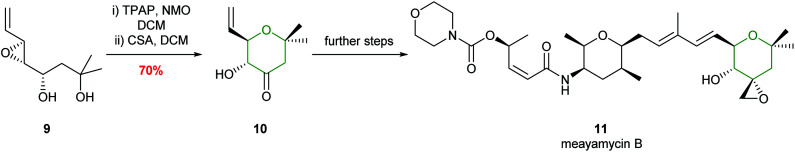
Total synthesis of meayamycin B by Koide *et al.*^32^

Unsaturated 4,5-epoxy alcohols have been widely used as intermediates in natural product synthesis.^25,26^ Examples include the preparation of mucocin, amphidinol 3 and the formation of the E ring in maitotoxin, as well as the botcinins, and more recently in the total synthesis of meayamycin B, illustrating the widespread value of the method (Scheme 3).^27–32^

**Scheme 4:**
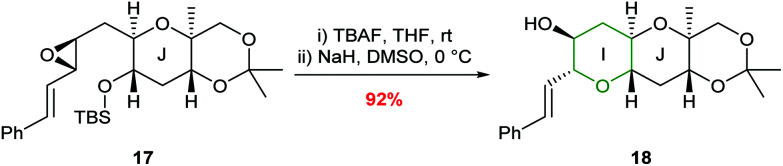
Synthesis of the I ring of brevetoxin B.^35^

In 1997, Nakata and co-workers reported that introduction of a styryl group adjacent to the epoxide provided greater stabilisation in the 6-*endo* transition state compared with the alkenic epoxides.^33,34^ Treatment of *trans*-epoxides containing a styryl group with either acid or base generated THP products in excellent yield, but improved stereocontrol was achieved using NaH (entries 1 and 2, Table 1). Whilst Nicolaou reported that unsaturated *cis*-epoxides gave poor 6-*endo* selectivity under acidic conditions,^23^ Nakata showed that styryl epoxides may be converted solely to THP products under acidic conditions, with no evidence for the formation of the corresponding THF (entries 3 and 4, Table 1). In contrast, under basic conditions *cis*-epoxides gave a mixture of 5- and 6-membered rings (entry 5). Isomerisation of the styryl double bond may occur in acid but in general this is not a problem as this directing group is often removed after cyclisation. Hence, although both *cis* and *trans*-epoxides with a styryl group can be cleanly cyclised, the need to remove the styryl directing group can be a disadvantage and as such, these substrates are only employed when the stabilisation of alkenic epoxides is insufficient to achieve the desired selectivity. For example, Nakata and co-workers used styryl-containing epoxides in their synthesis of the IJK ring system of brevetoxin B and the total synthesis of hemibrevetoxin B (Scheme 4).^35,36^

**Table 1:** Cyclisation of hydroxy styryl epoxides^33^

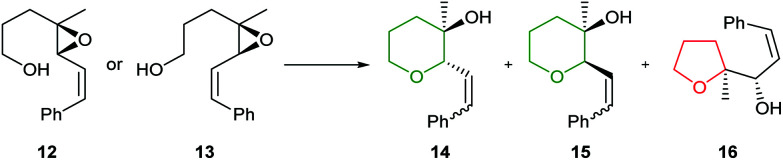				
Entry	Epoxide	Conditions	Yield	14 : 15 : 16
1	12	CSA (0.2 eq.), DCM, −78 °C, 1 h	100%	90 : 10 : 0
2	12	NaH (10 eq.), DMSO, rt, 2.5 h	97%	100 : 0 : 0
3	13	CSA (0.2 eq.), DCM, rt, 10 min	85%	30 : 70 : 0
4	13	AcOH–H_2_O (10 : 1), rt, 19 h	62%	15 : 85 : 0
5	13	NaH (20 eq.), DMSO, rt, 19 h	77%	0 : 66 : 34

**Scheme 5:**
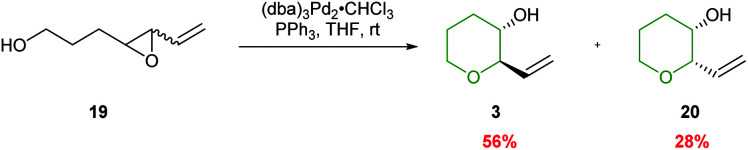
Palladium catalysed cyclisation of 4,5-epoxy alcohols.^37^

Both palladium and rhodium catalysts have been successfully employed in selective 6-*endo* ring closures of 4,5-epoxy alcohols. Pioneering work by Trost demonstrated that activation of vinyl epoxides could lead to exclusively the 6-*endo* products, albeit with poor stereoselectivity (Scheme 5).^37^ The reaction proceeds *via* initial formation of a π-allylpalladium intermediate, which in turn is trapped by the appended alcohol. The formation of the π-allylpalladium intermediate ensures only the THP is formed.

Through modification of the substrate, Hirama and co-workers converted both *cis* and *trans*-epoxides to the corresponding *anti* and *syn*-products in excellent yields and stereoselectivities (Table 2).^38^*In situ* formation of an ammonium alkoxide nucleophile alongside the use of an α,β-unsaturated ester to replace the alkene were vital for achieving the desired transformation.

**Table 2:** Improved palladium catalysed cyclisation of 4,5-epoxy alcohols^38^

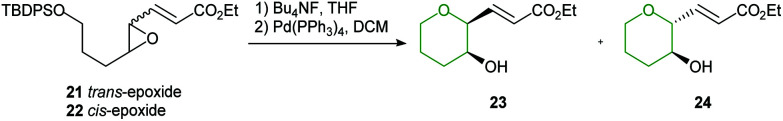				
Entry	Substrate	Reaction time	Yield	23 : 24
1	21	5 min	90%	>99 : 1
2	22	6 min	86%	2 : 98

**Scheme 6:**
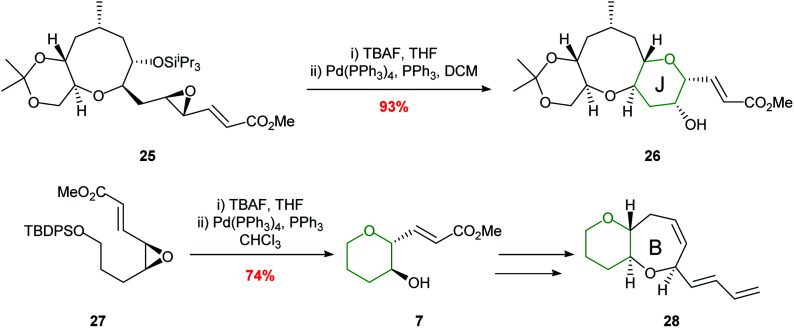
Synthesis of the J ring of ciguatoxin (top) and B ring of gambiertoxin 4B (bottom).^39,40^

Hirama employed a palladium-mediated cyclisation in the synthesis of the HIJ ring system of ciguatoxin and the AB ring fragment of gambiertoxin 4B (Scheme 6).^39,40^ The key cyclisations proceeded smoothly in 93% and 74% yields respectively to afford single diastereoisomers.

**Scheme 7:**
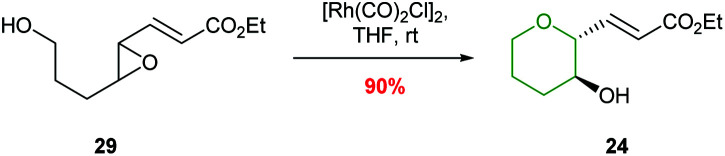
Rhodium catalysed intramolecular epoxide ring opening.^41^

Ha and co-workers expanded the scope of transition metals by using rhodium catalysis to promote selective THP formation (Scheme 7).^41^ Stirring *trans*-epoxide 29 with [Rh(CO)_2_Cl]_2_ in THF at room temperature gave *anti*-THP 24 with overall retention of stereochemistry *via* a double inversion mechanism. Interestingly, *cis*-epoxides were unreactive under these conditions. This was proposed to be due to a steric clash preventing the formation of the required π-allylrhodium species.

**Scheme 8:**
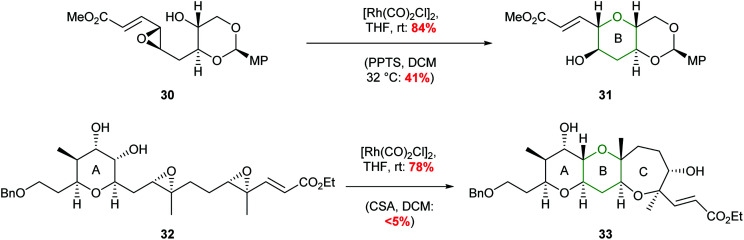
Synthesis of B ring of ciguatoxin C (top) and BC rings of (−)-brevisin (bottom). MP = 4-methoxyphenyl.^42,43^

The value of this methodology was demonstrated during the synthesis of the ABCDE ring fragment of ciguatoxin C. Hirama reported that cyclisation of acetal 30 under acidic conditions led to some deprotection of the acetal resulting in the required product 31 being isolated in only 41% yield (Scheme 8).^42^ In contrast using mild rhodium catalysed conditions, the required product 31 was isolated in 84% yield. Furthermore, in 2015 Jamison reported the creation of the B and C rings of (−)-brevisin through a cascade process under rhodium catalysis, whereas acidic conditions gave the required product 33 in low yield (Scheme 8).^43^

#### 2.1.2 Acetylenic epoxides

**Scheme 9:**
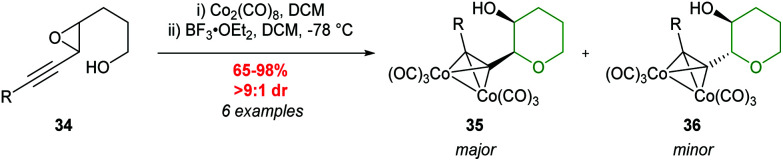
Cyclisation of racemic *trans*-acetylenic epoxides.^44^

**Scheme 10:**
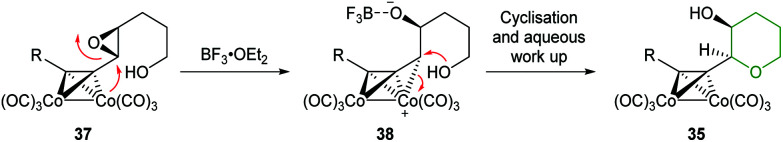
Mechanism for regioselective cyclisation of 4,5-epoxy alcohols 37.^45^

Another class of substrates which have been used to control cyclisation through cationic stabilisation are acetylenic epoxides. Hanaoka and co-workers reported that complexing acetylenic epoxide 34 with dicobalt octacarbonyl, followed by treatment with BF_3_·Et_2_O afforded exclusively THP products (Scheme 9).^44^ The alkynes can subsequently be restored by reaction with cerium ammonium nitrate (CAN). This adaption of the Nicholas reaction proceeds with retention of configuration at both epoxide carbons *via* a double inversion of the propargylic centre (Scheme 10).^45^ Both *cis* and *trans*-epoxides gave THPs in excellent yields and stereoselectivity, affording *anti* and *syn* products respectively. A variety of substituents on the alkyne can be used including silyl, alkyl, and aryl. It was proposed that the 6-*endo* product (THP) is formed in preference to the 5-*exo* product (THF) due to neighbouring group assistance of the cobalt complex (Scheme 10).

**Scheme 11:**
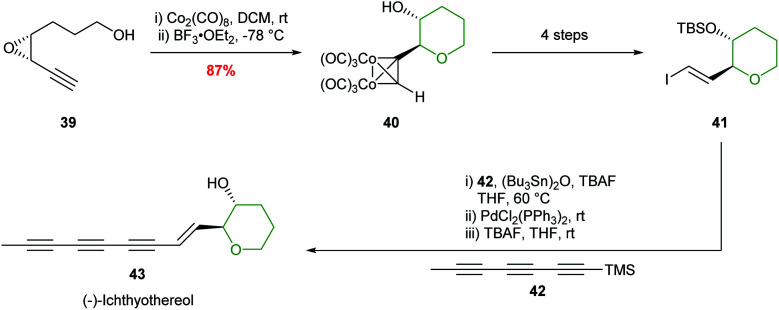
Total synthesis of (−)-ichthyothereol 43.^46^

Hanaoka and co-workers adopted this chemistry to complete the first total synthesis of (−)-ichthyothereol, where cobalt complexation followed by 6-membered ring formation proceeded in 87% yield and with excellent stereocontrol (Scheme 11).^46^

The use of acetylenic epoxides as substrates for the synthesis of oxygen heterocycles was further extended by Hanaoka and co-workers (Table 3).^47^ Both *cis* and *trans*-acetylenic epoxides with electron donating substituents on the alkyne were cleanly converted to the corresponding THPs through activation with BF_3_·Et_2_O, in the absence of the cobalt complex (entries C and D, Table 3). This is analogous to the results of Nicolaou (Scheme 2) whereby inversion of stereochemistry at the propargylic position is observed.^23^ In contrast, substrates containing electron withdrawing or electron neutral groups on the alkyne gave predominantly the 5-*exo* products.

**Table 3:** Alternative procedure for cyclisation of acetylenic epoxides 34^47^

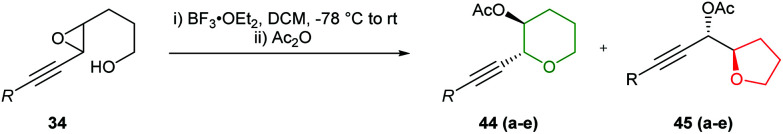			
Entry	R	Yield	44 : 45
A	H	92%	10 : 90
B	TMS	91%	62 : 38
C	*n*Bu	96%	95 : 5
D	Ph	94%	100 : 0
E	Bz	96%	1 : 99

**Scheme 12:**
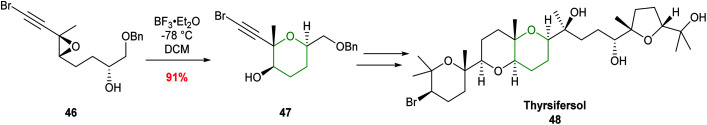
Total synthesis of thyrsiferol by Forsyth.^48^

Forsyth showcased the value of this procedure in 2000 during the total synthesis of thyrsiferol (Scheme 12).^48^ Cyclisation of trisubstituted epoxide 46 with BF_3_·Et_2_O gave THP 47 in 91% isolated yield and with complete regiocontrol.

In further studies, Hanaoka *et al.* illustrated that trisubstituted epoxides with an additional methyl group at either end of the epoxide (*i.e.* propargylic or homopropargylic positions) also led to 6-*endo* cyclisation using either the cobalt or Lewis acid mediated protocols.^49^ However, poor stereoselectivity was often observed for these classes of substrates using either procedure.

**Scheme 13:**
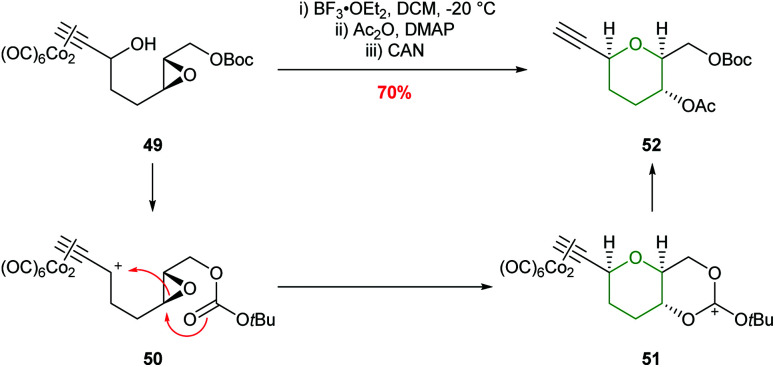
Intramolecular Nicholas reaction developed by Martín.^50^

In 2004, the synthesis of THPs using an intramolecular Nicholas reaction was reported by Martín *et al.* (Scheme 13).^50^ Activation of a dicobalt complexed propargylic alcohol 49 with BF_3_·Et_2_O generates carbocation 50 which is trapped by the oxirane oxygen. Although this process does not involve intramolecular epoxide ring opening of an acetylenic epoxide, but instead reversed reactivity of an epoxide attacking the propargylic center, the formal 6-*endo* product is still formed.

#### 2.1.3 Epoxysilanes

**Scheme 14:**
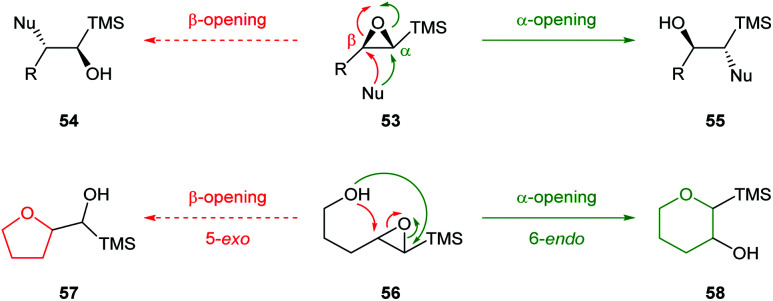
Inter and intramolecular nucleophilic ring opening of epoxy silanes 53 and 56.^51^

Epoxysilanes 53 are readily synthesised by epoxidation of vinyl silanes and undergo nucleophilic ring opening α to silicon (Scheme 14).^51^ This is in contrast to classical reactivity where silicon directs nucleophilic attack to the β-position due to stabilisation of the resultant positive charge through hyperconjugation. Computational studies by Paquette and Tomoda indicated the origin of α-opening is weakening of the σ_C–O_ bond α to silicon.^52,53^ The groups of Schaumann and Jamison have investigated the use silicon to direct intramolecular epoxide ring opening of 4,5-epoxy alcohols (Scheme 14).

**Scheme 15:**
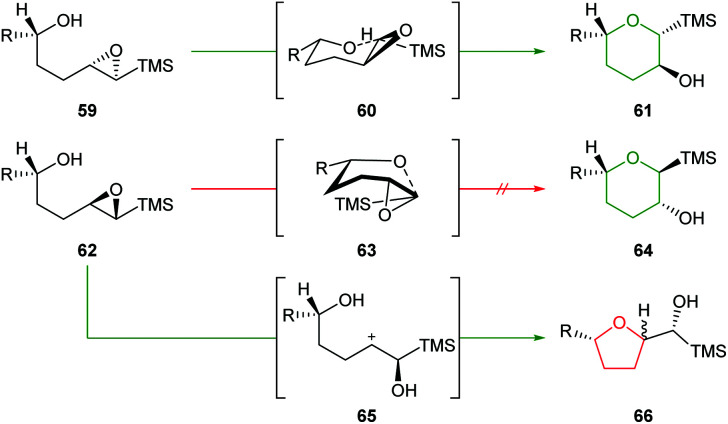
Intramolecular ring opening of 1,4-*anti* (top) and 1,4-*syn* (bottom) epoxy alcohols.^54^

Initially, Schaumann *et al.* conducted acid catalysed cyclisations of *trans*-4,5-epoxy alcohols which showed that 1,4-*anti* diastereoisomers 59 afforded the 6-*endo* products 61 whereas 1,4-*syn* diastereoisomers 62 yielded two epimeric 5-*exo* products 66 (Scheme 15).^54^ These results were rationalised by the ability of the 1,4-*anti* diastereoisomers to adopt a chair-like conformation 60 in the transition state whilst cyclisation of 1,4-*syn* diastereoisomers would require a boat-like transition state 63. To avoid this high energy pathway, the reaction instead could proceed *via* an S_N_1 mechanism, facilitated by the β-cation stabilisation effect of silicon.

**Scheme 16:**
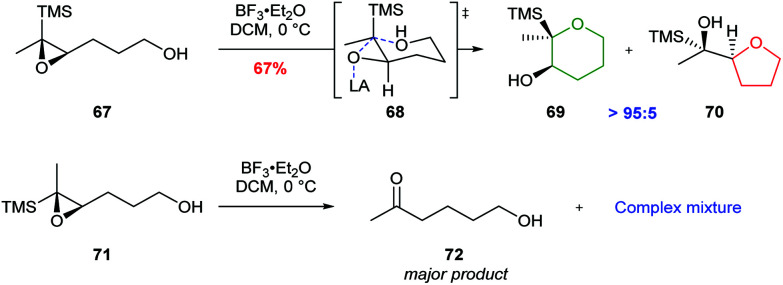
Cyclisation of *cis*-epoxysilane 67 and *trans*-epoxysilane 71 by Jamison.^55^

Jamison further investigated cyclisations of epoxysilanes as a strategy for THP synthesis in ladder polyethers.^55^ In this case, trisubstituted epoxysilane 67, which places silicon in the axial position during cyclisation, gave THP 69 as the major product, whereas the diastereomer 71 produced a complex mixture of products (Scheme 16). The use of epoxysilanes as substrates is of particular value for the selective synthesis of THPs as the silyl directing group may be readily removed *via* a TBAF promoted Brook rearrangement.

**Scheme 17:**
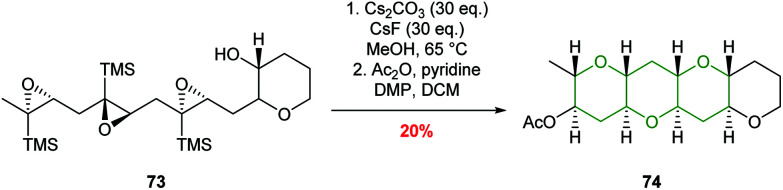
Epoxide-opening cascade directed by silicon.^56,57^

Jamison used this chemistry to good effect in iterative cascades, creating fused THP rings in excellent stereoselectivities and yields (Scheme 17).^56,57^ It was found that while BF_3_·Et_2_O was effective in the synthesis of isolated tetrahydropyrans, poor selectivity was obtained in cascade reactions. In contrast, the use of mildly basic conditions (Cs_2_CO_3_ and CsF in MeOH) facilitated the formation of fused THPs with concomitant deprotection of the silyl directing groups *via* a homo-Brook rearrangement (Scheme 17).

**Scheme 18:**
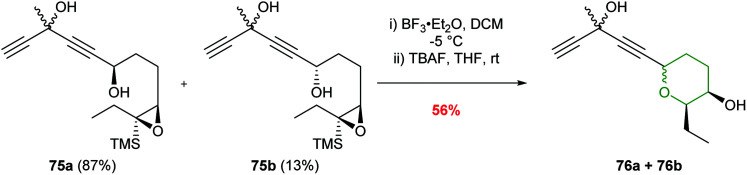
Synthesis of eastern THP ring of the jerangolids by Jauch.^58^

Jauch and co-workers used BF_3_·Et_2_O in the conversion of epoxysilane 75 to create the 6-*endo* product in 81% yield (Scheme 18).^58^ The silyl directing group was subsequently removed with TBAF to furnish THP 76 required for the construction of the jerangolids.

#### 2.1.4 Epoxysulfones

**Scheme 19:**
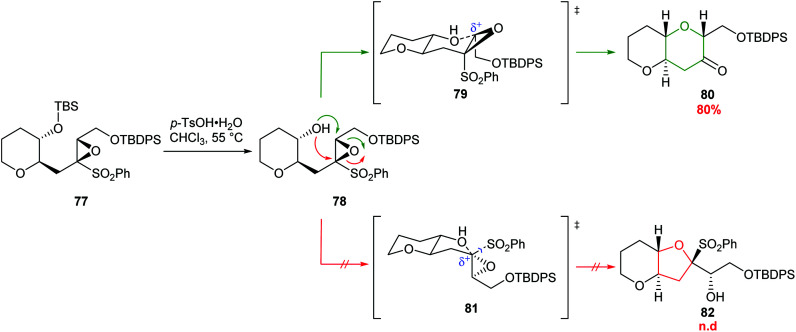
6-*endo* cyclisation of epoxysulfone 77.^59^

The use of alkenic and acetylenic epoxides as well as epoxysilanes as substrates for IERO all deliver the THPs as the major products by stabilising the 6-*endo* transition state. An alternative approach was developed by Mori, where judicious incorporation of a sulfonyl functional group on epoxide 77 led to destabilisation of the 5-*exo* transition state 81 and therefore formation of the 6-*endo* product 80 was favoured (Scheme 19).^59,60^ Upon treatment of epoxysulfone 77 with Brønsted acid, a sequence of silyl deprotection, 6-*endo* cyclisation and finally loss of phenylsulfonate afforded THP 80 in 80% yield. This process cleanly removes the sulfonyl directing group *in situ* and generates a ketone which can serve as a valuable functional group for further synthetic manipulations. The strong electron withdrawing nature of the sulfonyl group disfavours nucleophilic attack α to sulfur, which would proceed *via* the high energy transition state 81, and 5-*exo* product 82 was not detected.

**Scheme 20:**
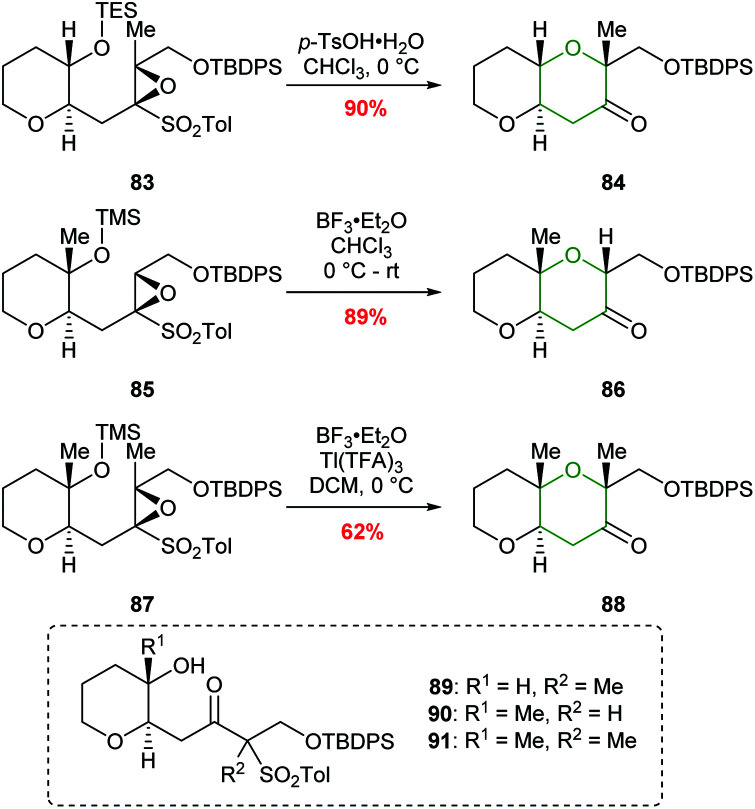
Conversion of methyl substituted epoxy sulfones 83, 85 and 87 to the corresponding THP products.^61^

A common structural feature of polyether marine toxins is the presence of methyl groups on *trans*-fused polytetrahydropyran ring systems. Mori constructed these scaffolds in excellent yields from epoxysulfones (Scheme 20).^61^ For example, treatment of epoxysulfone 77 with *p*-TsOH at 55 °C gave THP 80 in 80% yield (Scheme 19). Alternative reaction conditions were required when the substrate possessed either a tetrasubstituted epoxide (83) or a silyl protect tertiary alcohol (85 and 87) rather than a secondary alcohol (Scheme 20). Treatment of epoxide 83 with TsOH at 0 °C gave 84 in 90% yield whereas at higher temperatures 1,2-sulfonyl migration occurred leading to ketone 89 as the major product. For tertiary silyl ether 85, Lewis acidic conditions were required to facilitate clean conversion to the THP 86. Finally, for the formation of dimethyl-substituted THP 88 from 87, BF_3_·Et_2_O was used to promote cyclisation, and Tl(TFA)_3_ was added to suppress the problematic 1,2-sulfonyl rearrangement.

**Fig. 4:**
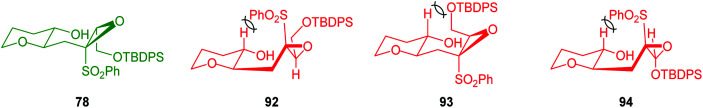
Proposed transition states and corresponding steric clashes for the intramolecular epoxide ring opening of epoxysulfones 78, 92, 93 and 94.^62^

**Scheme 21:**
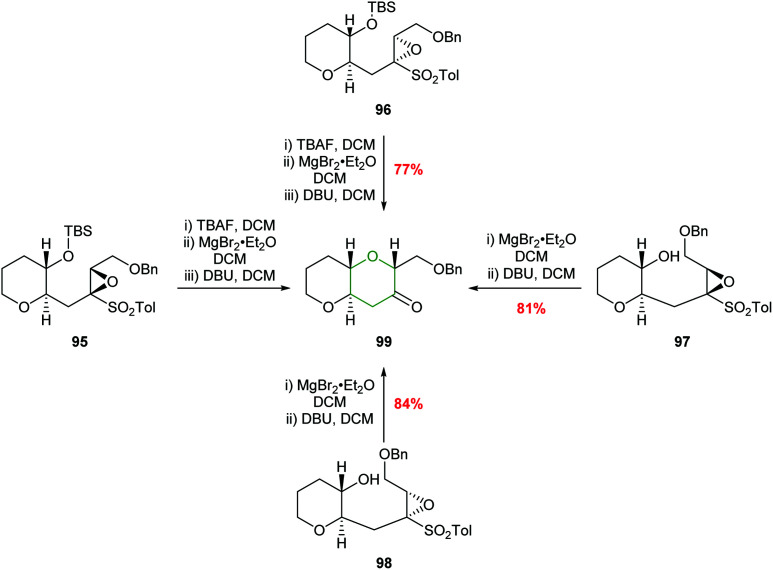
Alternative procedure for the synthesis of THP 99.^62^

The relative stereochemistry of the epoxysulfone substrate proved to be important in the outcome of reactions with Brønsted acids with cyclisation only occurring with substrates such as 78, whilst 92, 93 and 94 were unreactive (Fig. 4). These differences in reactivity were proposed to be due to steric interactions preventing the unreactive isomers adopting the 6-*endo* transition states.^62^ To expand the substrate scope, Mori used alternative reaction conditions to effect cyclisation and deliver THPs (Scheme 21). Thus, the silyl ether of the epoxysulfone (95 or 96) was first deprotected with TBAF and the resultant alcohols treated with MgBr_2_·Et_2_O and finally addition of DBU gave bicyclic ketone 99 in good yield and excellent stereocontrol.^62^ Although this is not an intramolecular epoxide ring opening process, the formal 6-*endo* product is obtained.

**Scheme 22:**
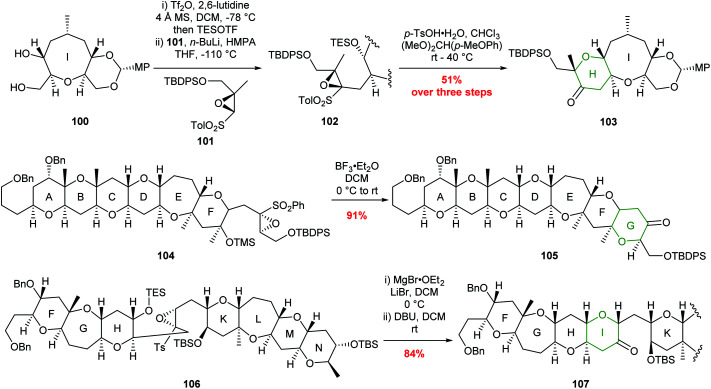
Examples of sulfonyl directed intramolecular epoxide ring opening in total synthesis. H ring of ciguatoxin (top), G ring of gambierol (middle) and I ring of gymnocin-A (bottom). MP = 4-methoxyphenyl.^65,66,68^

Sulfonyl directed intramolecular epoxide ring opening has been widely used by Mori and others to prepare THP containing natural products and some examples are shown in Scheme 22.^63–68^

#### 2.1.5 Trialkyl substituted epoxides

**Scheme 23:**
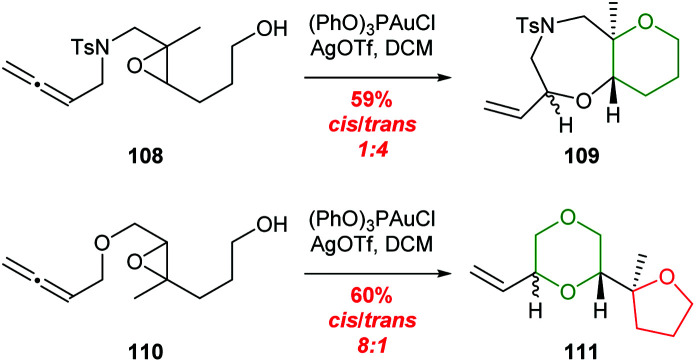
Gold-catalysed cyclisation of allenic epoxides 108 and 110.^72^

The ability of methyl groups to stabilise positive charged intermediates of epoxide ring-opening reactions in the synthesis of polycyclic polyethers has been reviewed previously.^21^ McDonald and co-workers reported that the nature of the terminating nucleophile plays an important role in the outcome of such cascade processes.^69–71^ Gagné *et al.* investigated the gold catalysed cyclisation of allenic epoxides 108 and 110 with methyl groups located at different ends of the trisubstituted epoxide (Scheme 23).^72^ By switching the position of the methyl group, 5-*exo* and 6-*endo* cyclisations occurred selectively.

**Scheme 24:**
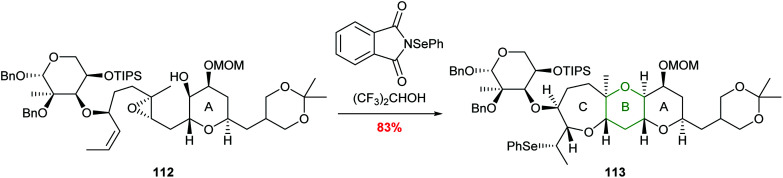
Synthesis of the B and C rings of hemibrevetoxin B.^73^

A further example of this regiocontrol was demonstrated by Holton in the total synthesis of hemibrevetoxin B reported in 2002 (Scheme 24).^73^ Reaction of 112 with *N*-(phenylseleno)phthalimide promotes a cascade process leading to formation of 6- and 7-membered oxygen heterocycles in 83% yield. The use of hexafluoroisopropanol (HFIP) as a solvent was proposed to be important to increase the charge separation in the reaction and therefore improve selectivity.

More recently, Qu and co-workers demonstrated how trialkyl substituted epoxides are converted to the corresponding THP products through reaction with 1-ethyl-3-methylimidazolium tetrafluoroborate ([EMIM]BF_4_) in perfluoro-*tert*-butanol (PFTB).^74^ In contrast, disubstituted epoxides formed solely the THF products. These contrasting results indicate the important role that an extra substituent on the epoxide can play in controlling 6-*endo* cyclisation. Many selective cyclisation cascades have been used to good effect to generate the core structures of marine polycyclic polyether natural products.

### 2.2 Reagent controlled formation of THPs from 4,5-epoxy alcohols

#### 2.2.1 TIPSOTf mediated cyclisations

**Scheme 25:**
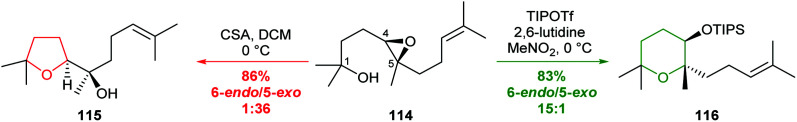
Reagent-controlled selectivity in cyclisations of 4,5-epoxy alcohol 114.^75^

Although controlling cyclisation using directing groups is effective, it imposes constraints on the substrates which can be used. It would be ideal to switch between the formation of the 5-*exo* and 6-*endo* products by simply altering the reaction conditions. This was elegantly demonstrated by Morimoto and co-workers during investigations into the synthesis of various oxasqualenoids (Scheme 25).^75,76^ Interestingly, despite the presence of a trisubstituted epoxide, Brønsted acid catalysed cyclisation of epoxy-alcohol 114 in DCM gave 5-*exo* product 115 in which attack occurred at the less substituted end of the epoxide. In contrast treatment of the same substrate 114, with TIPSOTf gave tetrahydropyran 116 in good yield. Morimoto proposed that the 6-*endo* product was formed preferentially due to steric repulsion between the bulky silyl group and the substituents on C-5 in the 5-*exo* transition state. Further investigations revealed that steric bulk at C-5 was required to maintain the preference of the 6-*endo* product. However, this process was limited to tertiary alcohols due to formation of triflates with other epoxy alcohols.

**Scheme 26:**
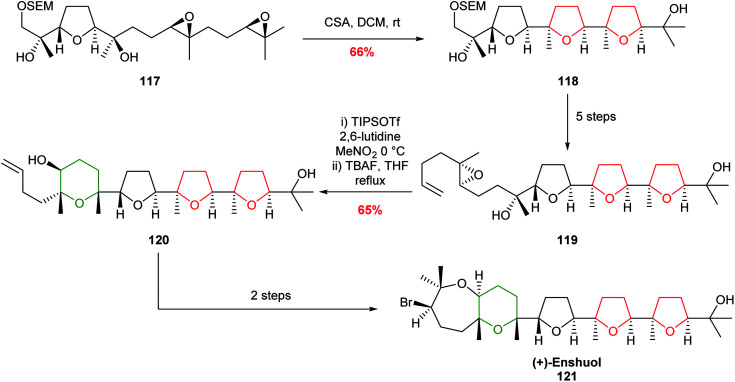
Total synthesis of (+)-enshuol by TIPSOTf promoted 6-*endo* cyclisation.^77^

In 2007, Morimoto demonstrated the value of these reagent-controlled cyclisations to create both 5 and 6 membered rings with excellent selectivity in the total synthesis of (+)-enshuol 121 (Scheme 26).^77^ Whilst this methodology still imposes constraints on the substrate structure, it demonstrates the power of using different reagents to control selectivity.

#### 2.2.2 Use of chiral phosphoric acid

**Scheme 27:**
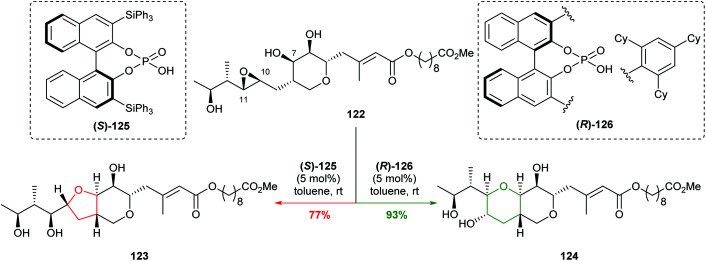
Cyclisations of epoxide 122 using chiral phosphoric acid catalysts.^78^

Pseudomonic acid A is an antibiotic produced by the bacterium *Pseudomonas fluorescens.* It is unstable under acidic conditions due to attack of the 7-hydroxyl group onto the 10,11-epoxide giving a mixture of bicyclic products (Scheme 27). In 2020, Nagorny and co-workers reported the reagent controlled cyclisation of methyl pseudomonate A 122 using chiral phosphoric acid catalysts to give either 123 or 124 in 77% and 93% yield respectively (Scheme 27).^78^

Through a combination of mechanistic and computational studies, Nagorny proposed the selectivity originated from steric clashes between the catalyst and substrate set up by a hydrogen bond network. In particular, steric interactions between (*R*)-126 and 122 increase the energy of the 5-*exo* transition state, whilst no such interactions occur between (*S*)-125 and 122. Although this is a substrate specific case, it elegantly illustrates the use of non-covalent interactions with a chiral catalyst in intramolecular attack on epoxides. With the aid of computational methods and experimental design, this work may pave the way forward for developing new catalysts for controlling cyclisation of a variety of 4,5-epoxy alcohols.

#### 2.2.3 Cobalt and vanadium catalysed cyclisations

**Scheme 28:**
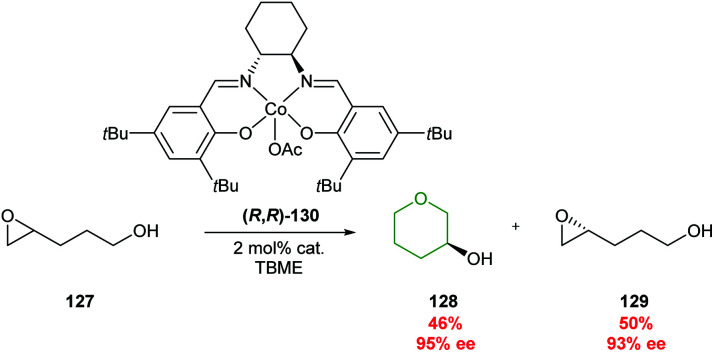
Kinetic resolution of 4,5-epoxy alcohols through cobalt catalysis.^79^

While the transition metal catalysed methods described earlier using palladium and rhodium required an α,β-unsaturated ester adjacent to the epoxide to facilitate 6-*endo* cyclisation (section 2.1.1), other more general metal catalysed cyclisations of 4,5-epoxy alcohols have been developed. In 1999, Jacobsen reported the use of Co^III^(salen) catalyst (*R*,*R*)-130 in the IERO of racemic epoxide 127, with complete 6-*endo* selectivity (Scheme 28).^79^ The chiral catalyst was selective for one enantiomer of epoxide 127, facilitating a kinetic resolution giving a separable mixture of THP 128 in 46% yield and 95% ee along with (*R*)-epoxide 129 (50% yield, 93% ee). The origin of this 6-*endo* selectivity was not discussed, although a mechanism for an analogous process has been proposed.^80^

**Scheme 29:**
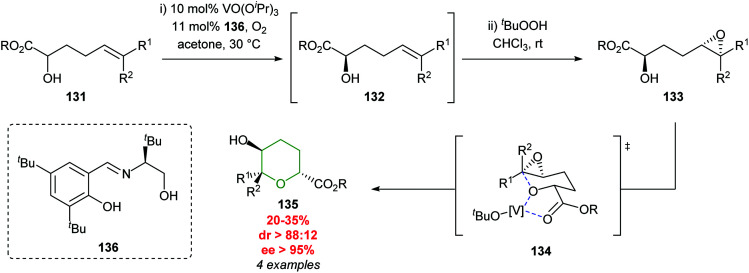
Vanadium catalysed synthesis of 2,5-*trans*-tetrahydropyrans.^81^

Later in 2006, Toste developed a vanadium catalysed kinetic resolution starting from unsaturated alcohols (Scheme 29).^81^ The approach proceeded *via* an initial resolution of α-hydroxy ester 131, followed by subsequent diastereoselective epoxidation of 132 and finally regioselective intramolecular epoxide ring opening to afford THP products 135. Cyclisation is proposed to proceed *via* coordination of the vanadium complex to the hydroxy ester 133*via* a chair like transition state 134.^4^ To the best of our knowledge, neither the cobalt or vanadium catalysed processes have been used in the total synthesis of natural products.

#### 2.2.4 Templated cyclisations in water

When investigating *endo*-selective epoxide ring opening cascades for the synthesis of marine polycyclic polyethers, Jamison proposed that regiocontrol may be achieved using a THP template within the starting material.^82^ Whilst poor selectivity was observed using Brønsted acids and bases as well as Lewis acidic conditions in organic solvents (entries 1–3, Table 4), when cyclisation was conducted in water, excellent 6-*endo* selectivity was achieved (entry 4).^83^ Qu and co-workers revealed that a combination of the aqueous environment and the template was required, as linear substrates afforded primarily THF products.^84^ Jamison *et al.* conducted detailed mechanistic studies whereby the role of the template and water was interrogated, confirming the role of the THP template.^85,86^

**Table 4:** Cyclisation of THP templated epoxy alcohol 137^83^

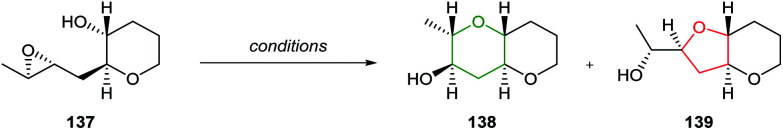		
Entry	Conditions	138 : 139
1	Cs_2_CO_3_, MeOH	1 : 2.7
2	BF_3_·Et_2_O, DCM	1.4 : 1
3	AcOH, toluene	1.6 : 1
4	H_2_O	>10 : 1

**Scheme 30:**
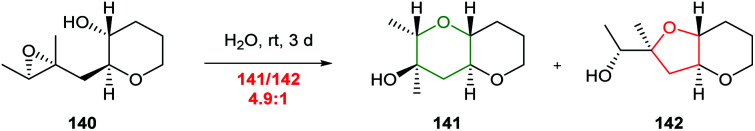
Water promoted 6-*endo* cyclisation of 4,5-epoxy alcohol 140.^70^

Whilst a methyl substituent on a trisubstituted epoxide can direct nucleophilic attack as described in section 2.1.5, interestingly Jamison demonstrated that cyclisation of THP templated substrates proceeded to give *endo* products such as 141 under aqueous conditions (Scheme 30).^70^

**Scheme 31:**
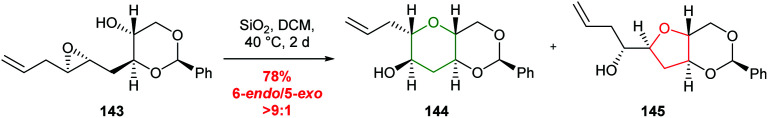
Silica gel promoted 6-*endo* cyclisation of benzylidene acetal templated epoxide 143.^87^

Building on the use of the THP ring template, Jamison developed a benzylidene acetal template to achieve selective 6-*endo* cyclisation which has the advantage that following reaction, the template was readily removed (Scheme 31).^87^ Both water and silica gel were used to promote the desired cyclisation.

**Scheme 32:**
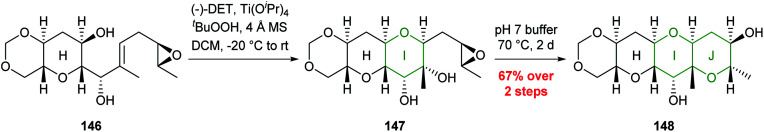
Synthesis of J ring of yessotoxin by Jamison.^88^

This methodology was used to good effect in the synthesis of the HIJK ring system of gymnocin A through a polycyclisation cascade in water, and the J ring of yessotoxin (Scheme 32).^87,88^

#### 2.2.5 Antibody catalysis

**Scheme 33:**
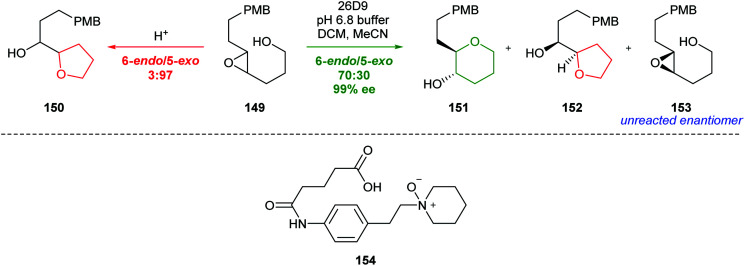
Cyclisation of epoxy alcohol 149 under acidic conditions and antibody catalysis (top) and transition state mimic 154 used to produce antibody catalyst 26D9.^89,91^

In 1993, Lerner *et al.* showed that antibodies could be used to catalyse the 6-*endo* cyclisation of 4,5-epoxy alcohol 149 (Scheme 33).^89^ A transition state mimic 154 was designed such that the anionic charge of the *N*-oxide would recruit cationic amino acid residues to promote epoxide ring opening, and cationic charge would induce the formation of anionic residues to stabilise a buildup of positive charge in the 6-*endo* transition state. Antibodies were produced in mice against the transition state mimic 154 and then purified. Of the 26 antibodies obtained, antibody 26D9 exhibited excellent catalytic activity, converting epoxide 149 to THP 151 as the major product. Importantly, antibody 26D9 was selective for one enantiomeric epoxide, resulting in a combined selective cyclisation and resolution. Computational studies indicated the antibody catalyst must lower the energy of the 6-*endo* transition state by more than 3.6 kcal mol^−1^ relative to the 5-*exo* transition state.^90^ Further studies by Lerner yielded the novel antibody 5C8, which also showed excellent catalytic activity. Through the use of X-ray crystallography and docking studies, an acid–base catalysed mechanism was proposed.^91^

A similar approach was investigated by Chiosis, but poor selectivity was achieved.^92^ Nevertheless, both these studies illustrate the power of antibody catalysis to overcome the inherent preference for 5-*exo* cyclisation of 4,5-epoxy alcohols.

#### 2.2.6 π-Anion catalysis

**Scheme 34:**
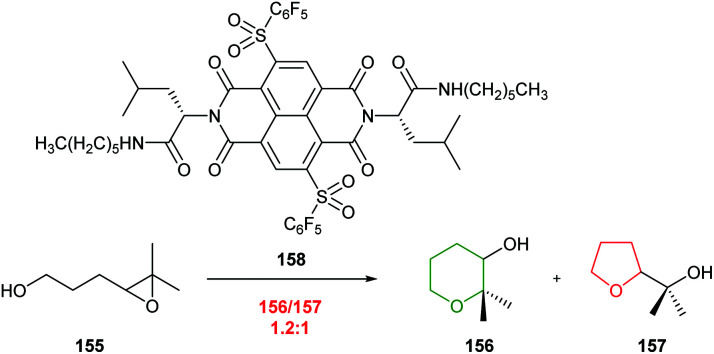
Cyclisation of 4,5-epoxy alcohol 155 catalysed by NDI 158.^93^

The recent development of strategies to stabilise anions through aromatic π-surfaces provides an exciting opportunity for controlling regioselectivity of reactions such as cyclisations of 4,5-epoxy alcohols.^93,94^ Matile and co-workers reported that π-anion surfaces can promote 5-*exo* cyclisation of 4,5-epoxy alcohols. Nevertheless, a slight preference for 6-*endo* cyclisation was observed when epoxide 155 was cyclised in the presence of naphthalene diimide (NDI) catalyst 158 (Scheme 34). Although the origin of this selectivity is unclear, this preliminary result illustrates the potential to develop alternative π-anion catalysts to overcome inherent 5-*exo* reactivity to form THP rings.

### 2.3 Further methods

#### 2.3.1 Expansion of THF rings

**Scheme 35:**
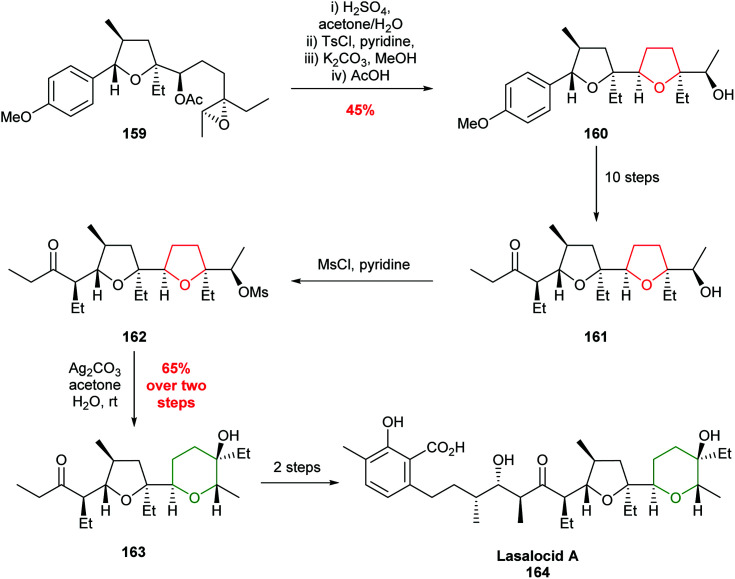
Total synthesis of lasalocid A.^95^

During the total synthesis of lasalocid A, Kishi revealed a valuable rearrangement which converted the 5-*exo* product, generated by an intramolecular epoxide ring opening, to the corresponding THP ring (Scheme 35).^95^ For example, following synthesis of THF 161, the secondary alcohol was converted to mesylate 162 and subsequent silver carbonate promoted ring expansion afforded THP 163 in 65% yield, which was used as an intermediate in the synthesis of lasalocid A. Whilst this strategy requires extra synthetic transformations, no directing group was required.

**Scheme 36:**
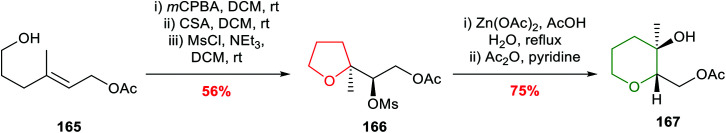
Preparation of THP 167 by ring expansion of 5-*exo* cyclisation product.^96^

Nakata and co-workers reported further investigations into this ring expansion and first converted unsaturated alcohol 165 to an epoxide which cyclised to the expected THF on treatment with camphorsulfonic acid in DCM (Scheme 36).^96^ The key ring expansion step was performed by heating mesylate 166 in AcOH and water in the presence of Zn(OAc)_2_ giving, after acetylation, THP 167 in 75% yield.

**Scheme 37:**
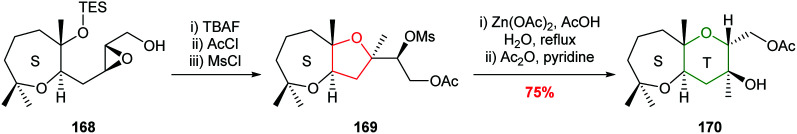
Synthesis of model ST rings of maitotoxin.^34,97^

This methodology was used in the synthesis of a model compound 170 with the ST rings of maitotoxin (Scheme 37).^34,97^

**Scheme 38:**
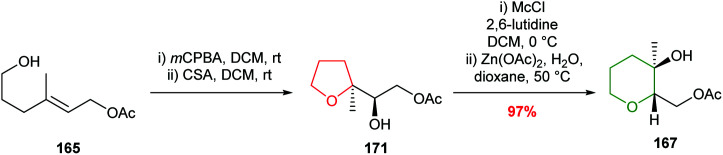
Alternative conditions for the rearrangement-ring expansion of 5-*exo* product 171. Mc = chloromethylsulfonate.^99^

Zinc acetate in refluxing acetic acid was required for the ring expansion of mesylates 166 and 169. However, Nakata showed that by using chloromethylsulfonate (Mc) as the leaving group, rather than mesylate, ring expansion was achieved without the need for acetic acid (Scheme 38).^98,99^ These modified conditions were used in the efficient conversion of THF 171 to THP 167. Furthermore, it was shown that ring expansion may be performed in the absence of a Lewis acid, albeit giving 6-membered rings in lower yield.

**Scheme 39:**
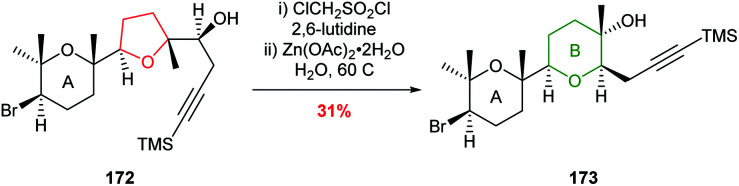
Synthesis of B ring in 15,28-dideoxy-15,28-didehydrothyrsenol.^100^

McDonald used this ring expansion strategy to create the B ring of 15,28-dideoxy-15,28-didehydrothyrsenol in 31% yield over 2 steps from alcohol 172 (Scheme 39).^100^

#### 2.3.2 Cyclisation of 4,5-epoxy-4-methoxymethyl-1-hexanols

**Scheme 40:**
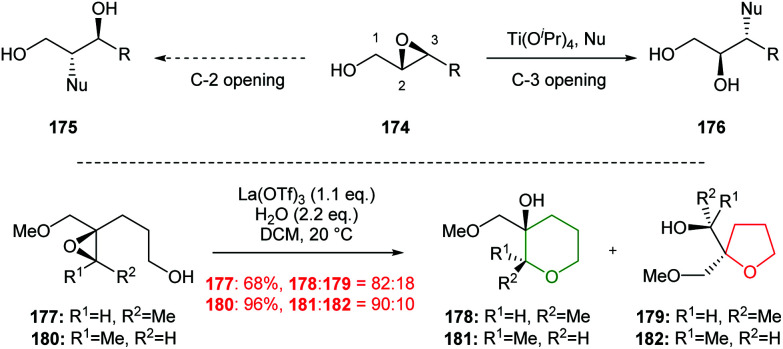
Inter- and intramolecular Lewis acid catalysed regioselective epoxide ring opening.^101,102^

The intermolecular reaction of nucleophiles with 2,3-epoxy alcohols in the presence of Ti(O^i^Pr)_4_ is an established method for achieving regioselective epoxide ring opening at the C-3 position (Scheme 40).^101^ Murai and co-workers investigated intramolecular variants, whereby chelation of 4,5-epoxy-4-methoxymethyl-1-hexanols 177 and 180 with Lewis acids would promote 6-*endo* over 5-*exo* cyclisation (Scheme 40).^102^ Optimisation studies revealed that La(OTf)_3_ with 2.2 equivalents (eq.) of water in DCM converted both *cis* and *trans* epoxides 177 and 180 to the corresponding THP products 178 and 181 respectively. These conditions proved to be highly specific, and changing solvent, water content or Lewis acid reversed the regioselectivity of epoxide ring opening or resulted in low yields. Murai proposed that the observed selectivity was due to water coordinating to La inducing an optimal chelation bite angle between the epoxide and methoxymethyl oxygen, which in turn leads to selective attack. However, no mechanistic or computational studies were reported to support this hypothesis.

**Scheme 41:**
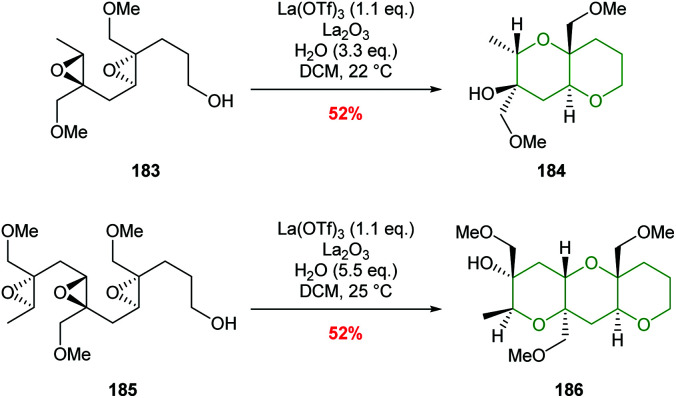
La(OTf)_3_ catalysed polyepoxide ring opening cascades.^103^

Two examples of the use of La(OTf)_3_ mediated cascade reactions have been reported leading to bicyclic and tricyclic products 184 and 186 (Scheme 41).^103^ There remains opportunities for exploitation of this methodology in natural product synthesis.

### 2.4 Summary of synthetic methods

In summary, many methods have been developed for the preparation of oxygen heterocycles from 4,5-epoxy alcohols. Structural features may be incorporated into the substrate, such as double and triple bonds, epoxysilanes and epoxysulfones, which favour formation of tetrahydropyrans *via* either stabilisation of a 6-*endo* transition state or destabilization of a 5-*exo* transition state, to afford tetrahydropyrans in high yields. These strategies have been used in natural product synthesis. There are exciting prospects for the use of reagents and catalysts in IERO to control 5-*exo* and 6-*endo* cyclisation of 4,5-epoxy alcohols. Both Lewis acids and chiral phosphoric acids have been used in regioselective cyclisations but to date these methods have not been widely used in the total synthesis of natural products. However, taking inspiration from nature might facilitate the optimisation of catalysts and development of novel methods for reagent controlled 6-*endo* cyclisation for THP formation. In the next section the biosynthesis of THPs *via* cyclisations of 4,5-epoxy alcohols are reviewed.

## 3. Biosynthetic methods

**Scheme 42:**
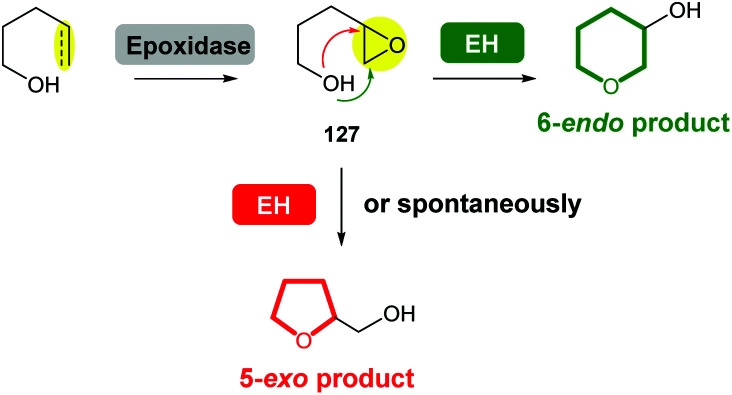
Biosynthesis of THPs and THFs *via* epoxide formation/epoxide-opening cascade reactions. EH: epoxide hydrolase.

Compared with the diversity of synthetic methods for the preparation of saturated oxygen heterocycles, a more limited set of reactions are used in natural product biosynthesis. However, epoxide formation/intramolecular epoxide-opening cascades are common processes used in the biosynthesis of many natural products. In some cases, epoxide hydrolases (EHs) have been identified which control the stereochemical outcome of cyclisation. When EHs are absent, labile epoxide biosynthetic intermediates 127 often spontaneously cyclise to generate 5-*exo* THF products (Scheme 42).

Interestingly, the biosynthesis of some natural products with structures containing THF rings has also been reported to be mediated by EHs, or in some cases, the 5-*exo* cyclisation is significantly accelerated by the presence of an EH. While this review focuses on THP formation, the biosynthesis of THF rings *via* a similar epoxide formation/epoxide-opening cascade reaction is topical and therefore is also discussed.

### 3.1 Formation of THPs from 4,5-epoxy alcohols catalysed by epoxide hydrolases (EHs)

#### 3.1.1 Lasalocid A

Lasalocid A isolated from *Streptomyces lasaliensis* is one of the important ionophore antibiotics among commercially available anticoccidial agents.^104^ Formation of the terminal THP ring in lasalocid A has attracted significant attention from researchers in the field and was amongst the earliest examples reported of intramolecular epoxide ring opening to generate a 6-membered ring in natural product biosynthesis.

**Scheme 43:**
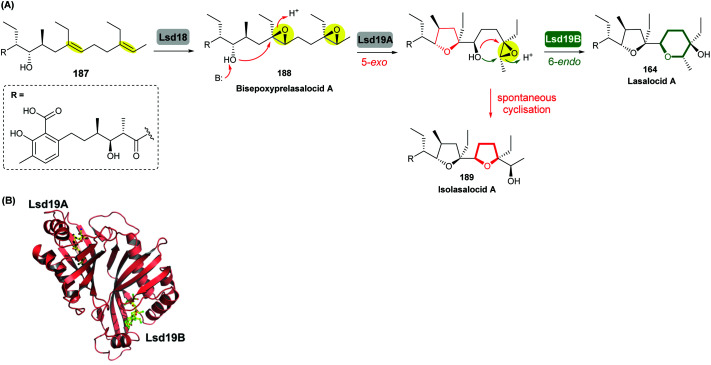
(A) THP ring formation in lasalocid A biosynthesis; (B) Crystal structure of Lsd19 with bound substrate (left half) and product (right half) analogues shown by stick models.^104–107^

Using *in vivo* and *in vitro* studies of the flavin-dependent monooxygenase (FMO) Lsd18, Minami *et al.* demonstrated that Lsd18 is responsible for oxidising the diene precursor 187 to afford bisepoxyprelasalocid A 188 (Scheme 43).^105^ In search for the enzyme responsible for selective epoxide-opening, Oikawa and co-workers identified Lsd19 as the putative epoxide hydrolase from the lasalocid biosynthetic gene cluster. *In vitro* turnover assays of bisepoxyprelasalocid A 188 with purified Lsd19 showed conversion of the synthetic substrate into lasalocid A (Scheme 43).^104^ In the absence of Lsd19, the synthetic bisepoxyprelasalocid A 188, which was obtained in only 3% yield, spontaneously cyclises to generate the THF ring of isolasalocid A 189.^104^ As discussed in section 2.3.1, Kishi synthesised the THP ring of lasalocid A by initial 5-*exo* cyclisation of an analogous epoxide 159 followed by ring expansion (Scheme 35). Whilst an elegant approach, this contrast between the synthesis and biosynthesis of the lasalocid THP ring highlights how nature can elegantly employ EH enzymes to readily overcome inherent selectivity for 5-*exo* cyclisation of 4,5-epoxy alcohols.

Concurrent *in vivo* studies by the Leadlay group on the targeted deletion of the *lsd19* gene (previously identified as *lasB*) in the lasalocid producing strain further supported the role of Lsd19 in directing biosynthesis towards lasalocid A rather than towards isolasalocid A.^106^ In the Δ*lasB* mutant of *Streptomyces lasaliensis*, production of lasalocid A was no longer detected, and isolasalocid A was the exclusive product.^106^

To gain a better understanding of how Lsd19 catalyses THP formation, Hotta *et al.* determined the X-ray crystal structure of Lsd19 in complex with its substrate and a product analogue to 1.59 Å resolution (Scheme 43).^107^ The epoxide hydrolase consists of two catalytic domains including the N-terminal region Lsd19A and the C-terminal region Lsd19B. The 5-*exo* and 6-*endo* epoxide-opening steps are catalysed by Lsd19A and Lsd19B, respectively. On the basis of structural analysis and quantum mechanical calculations, a mechanism of general acid–base catalysis has been proposed in which an aspartate residue acts as a base that activates the hydroxyl group on the substrate for a nucleophilic attack on the epoxide carbon, while a tyrosine residue acts as an acid to stabilise the negative charge that develops on the epoxide oxygen during the transition state of the reaction.^107^

#### 3.1.2 Aurovertin

Aurovertins are polyketides isolated from fungal species such as *Calcarisporium arbuscula* that exhibit significant inhibition of oxidative phosphorylation.^108^ Structurally, aurovertins contain an unusual dioxabicyclo-octane ring that is formed *via* iterative epoxidations and epoxide-openings of a polyene precursor.

**Scheme 44:**
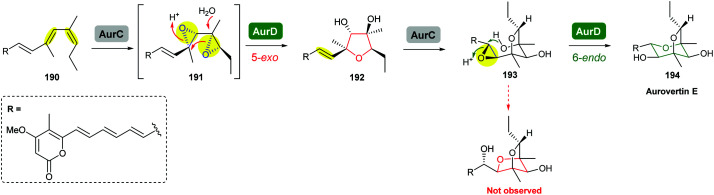
Proposed late-stage biosynthesis of aurovertins.^108^

The terminal triene portion 190 of the polyene precursor firstly undergoes bisepoxidation catalysed by the flavin-dependent monooxygenase AurC to form a proposed bisepoxide 191, which is then transformed into an isolable THF intermediate 192 through a 5-*exo* epoxide-opening step catalysed by the epoxide hydrolase AurD (Scheme 44). This stable THF intermediate then undergoes another round of epoxidation (by AurC) to give 193 and in this case cyclisation occurs *via* 6-*endo* ring opening (by AurD) to yield the dioxabicyclo-octane ring product 194.^108^

#### 3.1.3 Mupirocin/thiomarinol

**Scheme 45:**
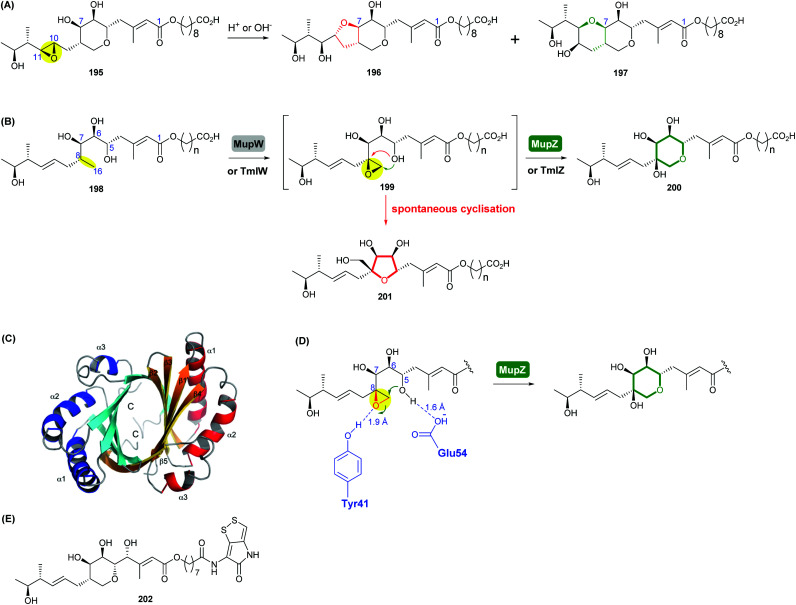
(A) Rearrangement products of pseudomonic acid A (195) formed under either acidic or basic conditions; (B) THP ring formation in mupirocin/thiomarinol biosynthesis; (C) crystal structure of MupZ; (D) proposed mechanism of MupZ-catalysed THP ring formation; (E) structure of thiomarinol A (202).^109,110^

Mupirocin, produced by *Pseudomonas fluorescens* NCIMB 10586, is a clinically important antibiotic against Gram-positive bacteria, including methicillin-resistant *Staphylococcus aureus* (MRSA).^109^ The major component of mupirocin (pseudomonic acid A, 195) bears a 10,11-epoxide which outside the pH range of 4–9, undergoes intramolecular attack by the 7-hydroxyl group to yield THF and THP rearrangement products 196 and 197 (Scheme 45).^109^ As discussed earlier in the synthetic methods section, Nagorny and co-workers recently reported the reagent controlled opening of this 10,11-epoxide to yield either the THF or THP as the major product (Scheme 27, section 2.2.2).^78^ However, of particular interest is the pharmacophoric THP ring of mupirocin, which is biosynthesised *via* a unique epoxide formation/epoxide opening cascade starting from a non-activated alkane precursor.^110^ Notably, all the other epoxidases reviewed herein use alkene precursors as substrates.

Using whole-cell biotransformations, Crump, Willis and co-workers showed that the C8–C16 single bond in the acyclic precursor 198 is oxidised by the Rieske non-haem oxygenase MupW to give the corresponding 8,16-epoxide 199 (presumably through an 8,16-alkene intermediate), which spontaneously cyclises giving a five-membered tetrahydrofuran ring product 201 (Scheme 45).^110^ When both MupW and the epoxide hydrolase MupZ are included in the biotransformation system, the hydroxylated THP ring product 200 is then generated. Deletion of the *mupZ* gene in the mupirocin producing strain abolished production of the THP ring metabolites and the THF ring compounds were produced instead. Based on X-ray crystallographic studies, molecular modelling and mutagenesis experiments of MupZ, the 6-*endo* epoxide-opening has been proposed to proceed through an acid–base mechanism, in which the Glu54 residue initially deprotonates the 5-hydroxyl group of the substrate (Scheme 45D), then the Tyr41 residue protonates the epoxide oxygen and subsequently stabilises the developing transition state, leading to the 6-*endo* THP ring product 200.^110^ This mechanism of general acid–base catalysis was found to be similar to that of the THP ring formation in lasalocid A biosynthesis.^107^

The thiomarinols, produced by marine bacteria such as *Pseudoalternomonas* sp. SANK 73390, are a group of natural products that closely resemble mupirocin and also show significant antibiotic activities against MRSA.^111^ Their structures are assembled on a highly equivalent polyketide backbone to mupirocin but are esterified by an 8-hydroxynonanoic acid chain and bear an additional non-ribosomal peptide synthase (NRPS)-derived pyrrothine moiety (Scheme 45E). TmlW/TmlZ are the MupW/MupZ equivalents in thiomarinol biosynthesis and each share over 55% sequence identity to their counterparts in the mupirocin pathway. Using the same whole-cell biotransformation approach, Wang *et al.* recently demonstrated that thiomarinol biosynthesis employs the same THP ring forming mechanism that requires the dual action of the Rieske non-haem oxygenase TmlW and the epoxide hydrolase TmlZ.^111^ It was also shown that these two pairs of enzymes are cross compatible between the mupirocin and thiomarinol systems.^111^

#### 3.1.4 Xiamenmycin

Xiamenmycins are benzopyran natural products isolated from *Streptomyces xiamenensis* 318 with antifibrotic and anti-inflammatory activities.^112^ Biosynthesis of the tetrahydropyran ring moiety in xiamenmycins requires the cooperation of a flavin-dependent monooxygenase (XimD) and a SnoaL-like cyclase (XimE).^112^

**Scheme 46:**
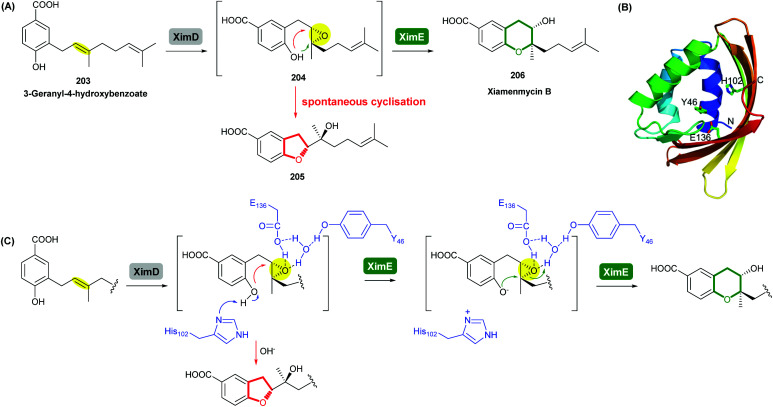
(A) THP ring formation in xiamenmycin biosynthesis; (B) crystal structure of XimE; (C) proposed mechanism of XimE-catalysed pyran ring formation.^112^

Using *in vitro* enzymatic assays, He *et al.* demonstrated that the 3-geranyl-4-hydroxybenzoate precursor 203 is converted by XimD to an unstable epoxide intermediate 204, which spontaneously transforms to a benzofuran product 205. Although the extra methyl substituent on the epoxide intermediate may have a directing effect that has been applied in synthetic efforts to favour the THP ring formation (see section 2.1.5 for examples), no THP ring product was reported in this case. However, when XimE was added in the assay, the 6-membered ring benzopyran xiamenmycin B 206 was formed as the major product (Scheme 46).^112^ Crystallographic structures of XimE, with and without product, suggested a synergistic mechanism in which residues E136, Y46, and H102 are functionally important to catalysis, substrate binding, and structural stabilisation (Scheme 46). Notably, in studies on substrate scope, these two enzymes exhibited high promiscuity capable of producing 14 products with 6 different benzoheterocyclic scaffolds.^112^ In a follow-up study, this pair of substrate-promiscuous enzymes were further employed for combinatorial biosynthesis of a series of benzoheterocyclic derivatives with greater structural diversity.^113^

### 3.2 Formation of THPs from 4,5-epoxy alcohols in which no EH has been identified

#### 3.2.1 Ambruticin

The ambruticins are a group of myxobacterial polyketides produced by various *Polyangium cellulosum* and *Sorangium cellulosum* strains with potent antifungal activities against a broad range of pathogens.^114^ Their structures feature a poly-olefinic skeleton, with an embedded trisubstituted cyclopropane and two oxygen heterocycles – a tetrahydropyran and dihydropyran.

**Scheme 47:**
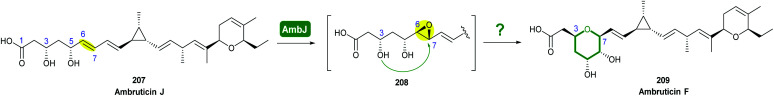
Proposed formation of the hydroxylated THP in ambruticin biosynthesis.^114^

The biosynthetic pathway was proposed by Reeves and co-workers through analysis of the biosynthetic gene cluster and characterisation of compounds produced by gene knockout strains.^114^ Disruption of the *ambJ* gene that encodes for a flavin-dependent epoxidase yielded ambruticin J lacking the THP ring. Thus, the THP ring formation was proposed to occur through epoxidation of ambruticin J 207 by AmbJ, followed by selective epoxide-opening of 208 at C-7 to give ambruticin F (Scheme 47). It is not known whether this cyclisation is under enzymatic or substrate control, but to-date no epoxide hydrolase has been identified in the ambruticin gene cluster.^114^ Nevertheless, as demonstrated by the work of Nicolaou (section 2.1), alkenic epoxides such as epoxy-ambruticin J 208 readily under IERO to form THP products due to stabilisation of the 6-*endo* transition state by the adjacent π-system.^22^ Our more recent studies involving cyclisation of model 4,5-epoxy alcohols are in accord with the proposal that both the 8,9-alkene and 5-alcohol of epoxy-ambruticin J 208 may play a role in controlling 6-*endo* cyclisation.^115^ In 2021 the total synthesis of ambruticin J was reported enabling further biosynthetic studies to be carried out to elucidate the role of AmbJ.^115,116^

#### 3.2.2 Abyssomicin

The abyssomicins are spirotetronate marine natural products isolated from various *Verrucosispora* and *Streptomyces* species.^117^ To date, ∼30 abyssomicins have been isolated and characterised, and they display a wide range of promising bioactivities including antibacterial and antiviral activities.^118^

**Scheme 48:**
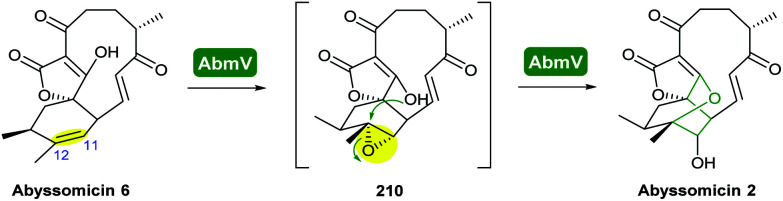
Proposed late stage pathway for abyssomicin biosynthesis.^118^

Combining *in vivo* gene inactivation with *in vitro* biochemical studies, Li *et al.* showed that the ether ring formation and hydroxylation in abyssomicin 2 biosynthesis is catalysed by the cytochrome P450 AbmV.^118^ This was proposed to result from a domino reaction sequence involving, (i) epoxidation of the 11,12-alkene in abyssomicin 6 to generate an epoxide intermediate 210 followed by, (ii) epoxide ring-opening *via* nucleophilic attack of the tetronate OH upon C12 to afford abyssomicin 2 (Scheme 48).

### 3.3 Formation of THFs catalysed by EHs

#### 3.3.1 Citreoviridin

Citreoviridin is a highly reduced polyketide product isolated from several *Penicillium* species. It has been shown to inhibit the mitochondrial ATP synthetase system.^119^ Citreoviridin has a close structural similarity to the aurovertins (see section 3.1.2) and THF ring formation in citreoviridin involves a bisepoxidation step to generate 211 followed by cyclisation similar to aurovertin biosynthesis.^108^

**Scheme 49:**
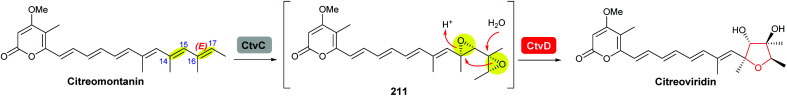
Proposed late stages biosynthesis of citreoviridin.^119^

Using *Aspergillus nidulans* as a heterologous expression host, Wang and co-workers reconstituted the pathway and demonstrated that four genes, ctvA, ctvB, ctvC, and ctvD, are sufficient to produce citreoviridin.^119^ Overexpression of the two genes ctvA and ctvB produced citreomontanin. In order to form the THF ring with the correct stereochemistry, the terminal alkene of citreomontanin with an *E*-16,17-alkene needs to undergo isomerisation to yield the *Z*-16,17 isomer, a step that could be catalysed by the flavin-dependent monooxygenase CtvC (Scheme 49). Bisepoxidation by CtvC then forms (17*R*,16*R*,15*S*,14*R*)-bisepoxide 211. Addition of ctvC to the overexpression system generated a mixture of new products, with citreoviridin being the major one among other unidentified products (possibly due to spontaneous hydrolysis and degradation of the unstable bisepoxide intermediate). Finally, when ctvD was added to generate the ctvABCD overexpression strain, the only observed product was citreoviridin (Scheme 49).^119^

#### 3.3.2 Ascofuranone

Ascofuranone is a meroterpenoid produced by various filamentous fungi including *Acremonium egyptiacum* and has been shown in recent studies to be a promising drug candidate against African trypanosomiasis and a potential anticancer lead compound.^120^

**Scheme 50:**
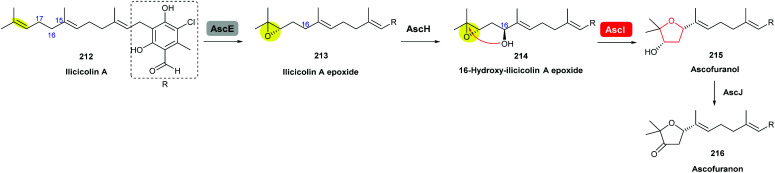
Proposed late-stage biosynthesis of ascofuranone.^120^

Using gene knockout experiments and *in vitro* enzymatic turnover assays, Araki *et al.* demonstrated that AscE is a P450 monooxygenase that catalyses stereoselective epoxidation of the terminal olefin of ilicicolin A to produce ilicicolin A epoxide 213, which is then hydroxylated by another P450 monooxygenase AscH to yield the 16-hydroxy-ilicicolin A epoxide intermediate 214 (Scheme 50). This epoxy alcohol 214 is then cyclised by AscI to form the THF ring in ascofuranol 215, and finally oxidation of the secondary alcohol to a ketone by AscJ delivers the product ascofuranone 216. It is interesting to note that the 16-hydroxy-ilicicolin A epoxide intermediate 214 can also be converted into ascofuranol nonenzymatically under acidic conditions. AscI shares 29% and 27% identities, respectively, to the epoxide hydrolases AurD and CtvD in aurovertin and citreoviridin biosynthesis (see previous sections).^120^

#### 3.3.3 Aurachin

Aurachins are a group of myxobacterial quinoline alkaloids produced by *Stigmatella aurantiaca* Sg a15 that exhibit antibacterial and antifungal among many other biological activities.^121^ The proposed late-stage biosynthesis of aurachin A involves epoxidation of the farnesyl side chain followed by an EH-catalysed epoxide-opening to form the THF ring product.^122^

**Scheme 51:**
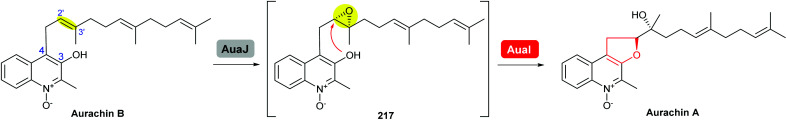
Proposed late-stage biosynthesis of aurachin A.^122,123^

Based on isotopic feeding studies, gene inactivation experiments and bioinformatic analysis, a biosynthetic sequence has been proposed for the late stage transformation of aurachin B to aurachin A (Scheme 51).^122,123^ The FAD-dependent monooxygenase AuaJ is believed to catalyse epoxidation of the 2′,3′-alkene on the farnesyl chain to give 217. Intramolecular attack of the epoxide intermediate 217 by the 3-hydroxyl would then result in the epoxide-opening and cyclisation to afford the THF ring. The proposed epoxide intermediate 217 has not been detected from the cell extracts, which may be due to rapid turnover. Although this process was proposed to be catalysed by an epoxide hydrolase, the function of AuaI remains to be confirmed by further studies.

#### 3.3.4 Penigequinolone/aspoquinolone

**Scheme 52:**
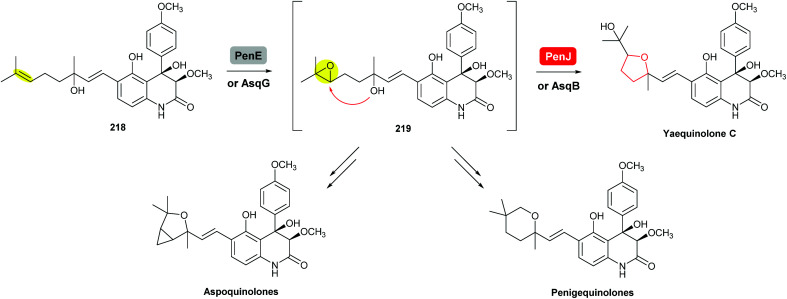
THF ring formation in yaequinolone C biosynthesis.^125,126^

Penigequinolones are insecticidal quinolone alkaloids produced by various *Penicillium* and *Aspergillus* species. Aspoquinolones are closely related analogues of penigequinolones and the two pathways share most of their biosynthetic machinery (Scheme 52).^124^

Yaequinolone C has been proven to be an off-pathway shunt product found during studies on penigequinolone biosynthesis.^125^ Formation of the THF ring in yaequinolone C has been shown by Zou *et al.* to result from epoxidation of the terminal olefin of the precursor 218 catalysed by the flavin-dependent monooxygenase PenE, followed by the PenJ-catalysed epoxide-opening of 219 to afford yaequinolone C (Scheme 52).^126^ It was found that yaequinolone C could also be formed when the alkene precursor 218 was incubated with only PenE, suggesting the epoxide-opening step could happen spontaneously. When the biotransformation was performed with both PenE and PenJ, the conversion of the precursor 218 to yaequinolone C was significantly elevated (∼10-fold), indicating the role of PenJ as an epoxide hydrolase in enhancing the rate of the 5-*exo* cyclisation step.^126^

The corresponding flavin-dependent monooxygenase/epoxide hydrolase pair AsqG/AsqB in the aspoquinolone pathway have been shown to be functionally equivalent in their ability to catalyse the same transformations.^125^ These two pairs of enzymes were also found to be cross compatible in mix-and-match experiments,^125^ which is similar to the mupirocin/thiomarinol case.^111^

In the presence of further enzymes, epoxide intermediate 219 undergoes further modifications to yield the final metabolites penigequinolones and aspoquinolones.^125^

#### 3.3.5 Monensin

**Scheme 53:**
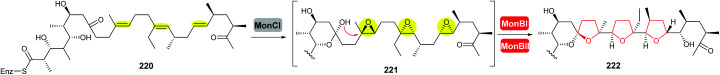
THF ring formation in monensin biosynthesis.^127,128^

Monensin A is a polyether ionophore isolated from *Streptomyces cinnamonensis* and has been widely used in veterinary medicine and in animal husbandry.^127^ Formation of the THF rings in monensin have been shown to proceed through epoxidation of an *E*,*E*,*E*-triene precursor 220 followed by an epoxide-opening cascade reaction (Scheme 53).

Deletion of the *monCI* gene, which encodes a putative flavin-dependent epoxidase from the monensin biosynthetic gene cluster, led to accumulation of an *E*,*E*,*E*-triene precursor 220 in mutant strains of *Streptomyces cinnamonensis*, suggesting MonCI is responsible for formation of the triepoxide intermediate 221.^127^ Subsequent epoxide-opening of the triepoxide intermediate was originally thought to be catalysed by MonCII but was later found to be controlled by the MonBI and MonBII epoxide hydrolases.^128^ Using structurally simple substrate analogues, Oikawa and co-workers demonstrated remarkable synergistic effect between MonBI and MonBII in catalysing epoxide-opening cascade reactions.^129^ When used alone in turnover reactions, MonBI was inactive and MonBII was only weakly active. However, the epoxide opening activity was dramatically enhanced with the addition of MonBI to the MonBII reaction mixture.^129^

Other epoxide hydrolases that have been proposed to be involved in THF ring biosynthesis include SalBI/SalBII and from salinomycin pathway,^130^ NigBI/NigBII from the nigericin pathway,^131^ NanI from the nanchangmycin,^132^ MadI from the maduramicin pathway,^133^ and Pak24/Pak25 from the K-41A pathway.^134^

### 3.4 Summary of biosynthetic methods

THP and THF rings are often essential structural moieties for bioactivities of many natural products and the formation of these oxygen heterocycles *via* epoxide formation/epoxide-opening cascade reactions has been demonstrated in a variety of bacterial and fungal natural product biosynthetic pathways. The epoxidation step is commonly catalysed by a flavin-dependent monooxygenase on an alkene substrate. However, interestingly in the mupirocin/thiomarinol systems, a Rieske non-haem oxygenase catalyses oxidation of a non-activated alkane precursor to an epoxide *via* an alkene. For regioselective intramolecular opening of epoxide biosynthetic intermediates, an epoxide hydrolase (EH) is often employed to yield the 6-*endo* cyclisation THP product. Only three protein structures (Lsd19, MupZ and XimE) have been reported for these EHs so far and X-ray crystallographic studies have suggested a similar mechanism of general acid–base catalysis. In the cases of ambruticins and abyssomicins where EHs have not been identified, further studies would be required to determine whether their THP ring formation steps are under enzymatic or substrate control. Cyclisation of 4,5-epoxy alcohols to generate THFs has been shown to either occur spontaneously or to be mediated and significantly accelerated by EHs, although no protein structure has been reported for this type of EHs yet. These epoxidation/epoxide-opening cascade reactions are summarised in Table 5.

**Table 5:** THP and THF ring formation *via* epoxidation/epoxide-opening cascade reactions in natural product biosynthesis

Natural product	Strain	Substrate	Epoxidase	Epoxide hydralase	Product	Ref.
Lasalocid A	*Streptomyces lasaliensis*	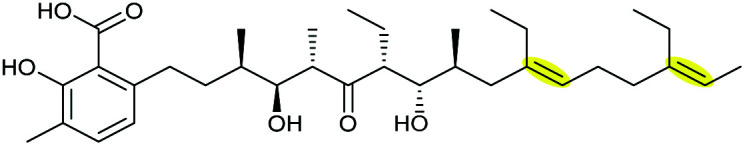	Lsd18 (FMO)	Lsd19 (PDB ID: 3RGA)	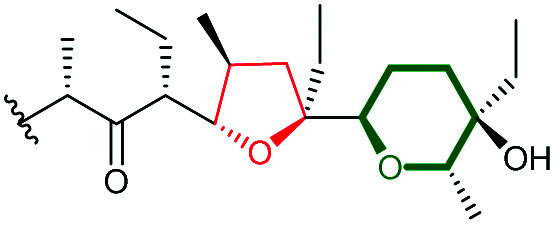	104–107
Aurovertin	*Calcarisporium arbuscula*	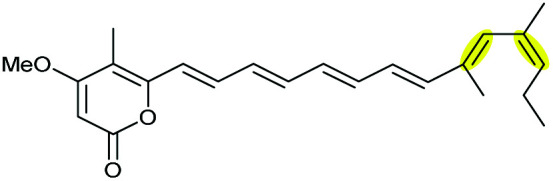	AurC (FMO)	AurD	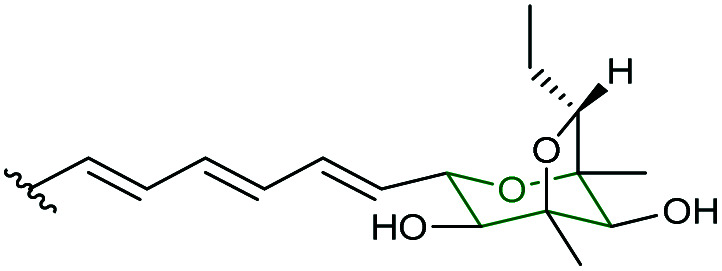	108
Mupirocin	*Pseudomonas fluorescens*	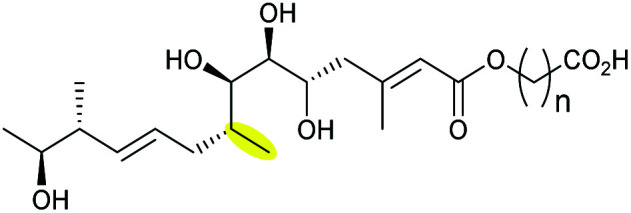	MupW (Rieske non-haem oxygenase)	MupZ (PDB ID: 6FXD)	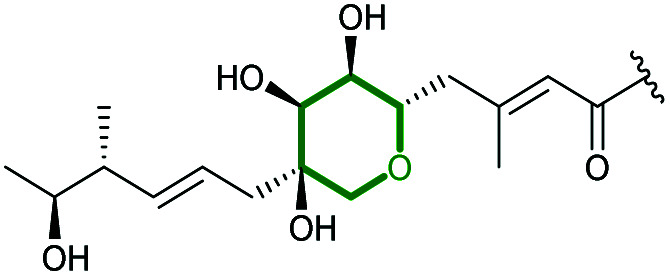	110
Thiomarinol	*Pseudoalternomonas* sp.		TmlW (Rieske non-haem oxygenase)	TmlZ		111
Xiamenmycin	*Streptomyces xiamenensis*	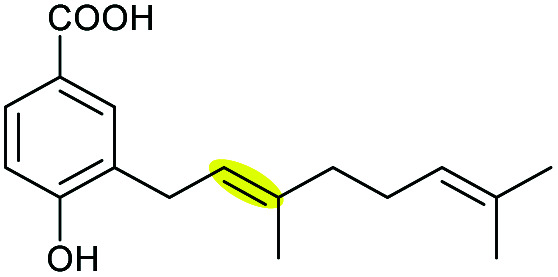	XimD (FMO)	XimE (PDB ID: 6ISK)	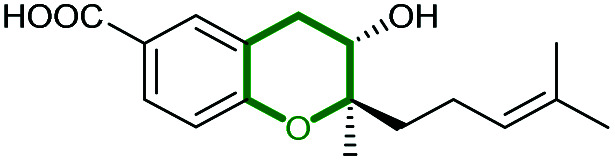	112
Ambruticin	*Sorangium cellulosum*	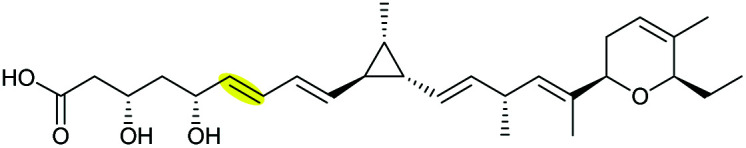	AmbJ (FMO)	Not reported	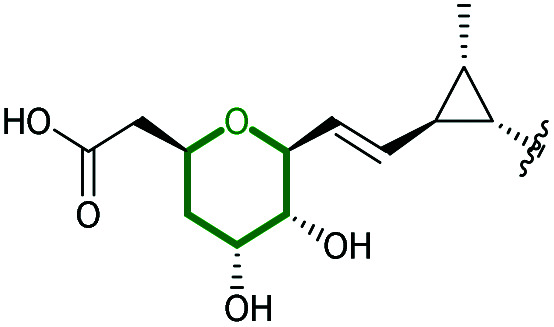	114
Abyssomicin	*Streptomyces koyangensis*	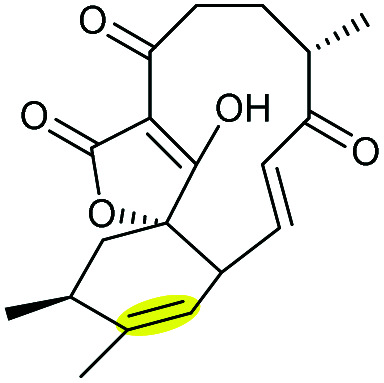	AbmV (P450)	Not reported	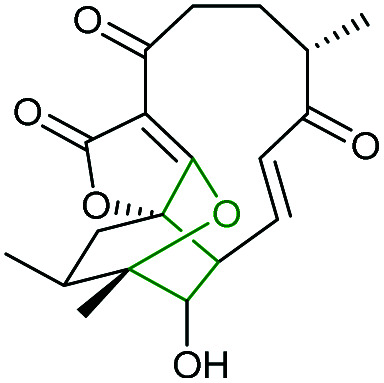	118
Citreoviridin	*Penicillium* sp.	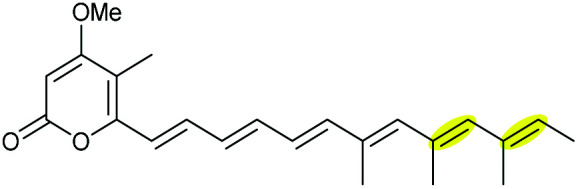	CtvC (FMO)	CtvD	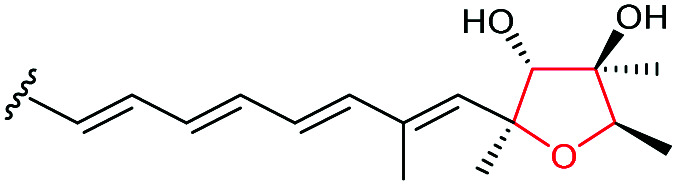	119
Ascofuranone	*Acremonium egyptiacum*	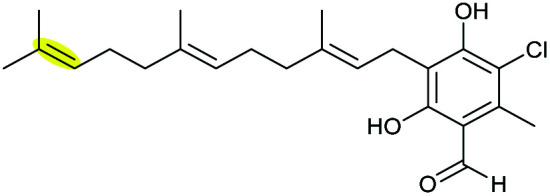	AscE (P450)	AscI	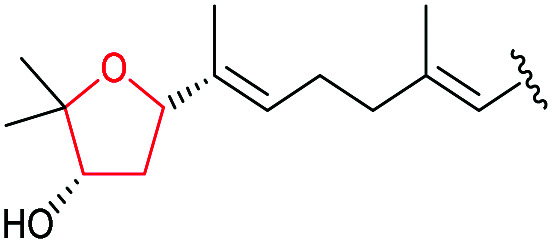	120
Aurachin	*Stigmatella aurantiaca*	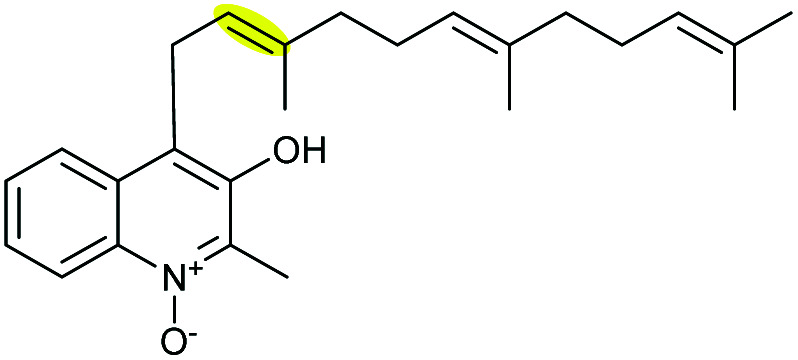	AuaJ (FMO)	AuaI	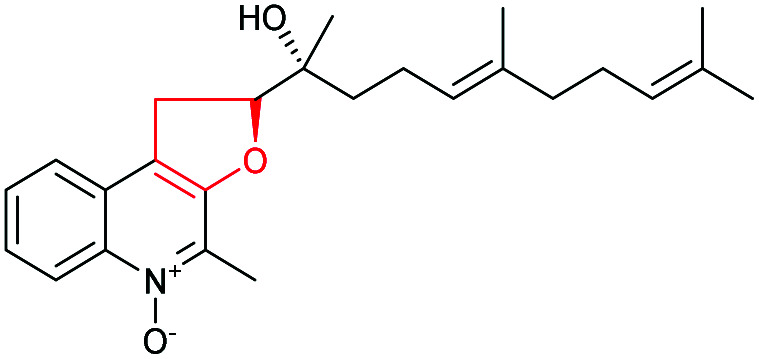	122
Penigequinolone	*Penicillium & Aspergillus* sp.	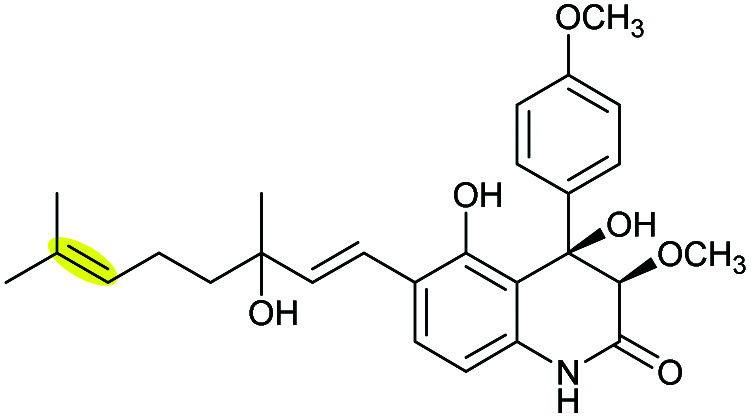	PenE (FMO)	PenJ	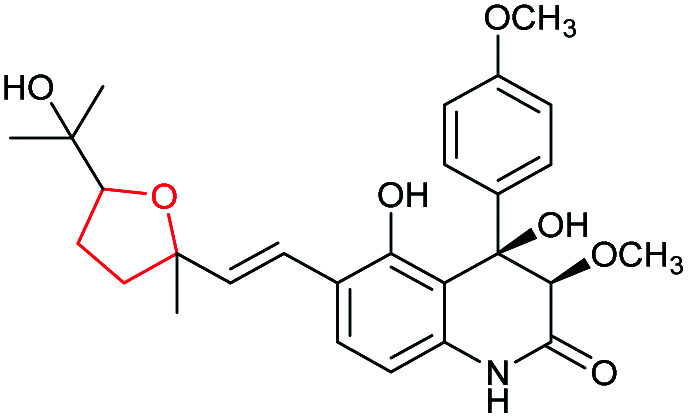	125 and 126
Aspoquinolone	*Aspergillus Nidulans*		AsqG (FMO)	AsqB		126
Monensin	*Streptomyces cinnamonensis*	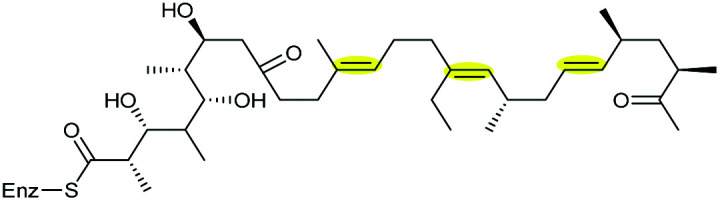	MonCI (FMO)	MonBI, MonBII	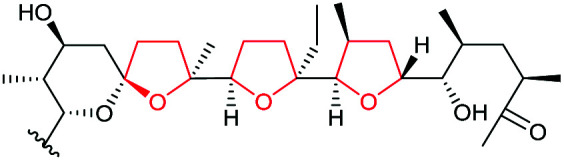	127–129

## 4. Future outlook

### 4.1 Combining chemical synthesis and biosynthesis

Despite advances in chemical methodology aided by computational studies, the total synthesis of complex natural products remains a challenging endeavour. Modular approaches to the total synthesis of target compounds often provide flexibility for the assembly of libraries of natural product analogues but may rely on the use of protecting groups, precious metals, toxic reagents and solvents whilst generating substantial amounts of unwanted by-products. On the other hand, enzyme-catalysed transformations offer clean and selective methods to generate target compounds but there can be issues to overcome in terms of narrow substrate specificities and scale-up. Hybrid strategies combining chemical synthesis and biotransformations continue to provide exciting new opportunities to gain efficient access to supplies of complex natural products and their analogues that are not accessible by individual synthetic or biotechnological approaches.^135^

### 4.2 Enzyme engineering

In a world with rising demands for clean, efficient, and selective catalysts, biosynthetic enzymes will continue to provide a repertoire of powerful tools to generate complex molecules and their derivatives. However, the catalytic potential of these enzymes is still far from fully explored.^136^

**Scheme 54:**
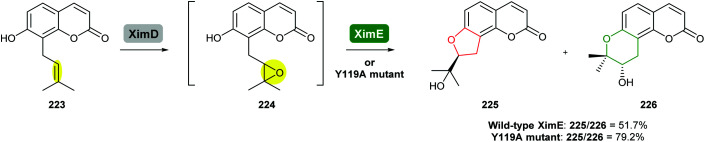
Formation of pyranocoumarins by engineered XimE.^113^

In a recent study, Xu and co-workers engineered XimE from xiamenmycin biosynthesis to improve its catalytic activity for the preparation of angular pyranocoumarins by site-directed mutagenesis (Scheme 54).^113^ Guided by the crystallographic structure of XimE and molecular docking, the Y119A mutant of XimE was generated. The proportion of THP product 226 to THF product 225 increased from 51.7% in wild-type XimE to 79.2% in the Y119A mutant.^113^

As further epoxide hydrolases are discovered, rational engineering of these enzymes to expand their substrate scope, enhance their catalytic activity and to possibly switch their regioselectivity in epoxide-opening process will be a fascinating area of research. For enzymes to be utilised in combination with organic synthesis, it will be necessary to optimise their stability, substrate scope and performance under a variety of conditions such as in the presence of organic solvents. We anticipate such efforts will tune and diversify the functions of these enzymes to provide biocatalysts that bring the benefits of nature's biosynthetic machinery to chemical synthesis.

## Conflicts of interest

There are no conflicts to declare.

## Supplementary Material
